# *miR-277* targets the proapoptotic gene-*hid* to ameliorate Aβ42-mediated neurodegeneration in Alzheimer’s model

**DOI:** 10.1038/s41419-023-06361-3

**Published:** 2024-01-18

**Authors:** Prajakta Deshpande, Chao-Yi Chen, Anuradha Venkatakrishnan Chimata, Jian-Chiuan Li, Ankita Sarkar, Catherine Yeates, Chun-Hong Chen, Madhuri Kango-Singh, Amit Singh

**Affiliations:** 1 Department of Biology, University of Dayton, Dayton, OH 45469 USA; 2 Institution of Molecular and Cellular Biology, National Taiwan University, Taipei, Taiwan; 3 National Institute of Infectious Diseases and Vaccinology, National Health Research Institutes, Miaoli, Taiwan; 4 Premedical Program, University of Dayton, Dayton, OH USA; 5 Integrative Science and Engineering (ISE), University of Dayton, Dayton, OH USA; 6 Center for Genomic Advocacy (TCGA), Indiana State University, Terre Haute, IN USA

**Keywords:** Alzheimer's disease, Cell death in the nervous system

## Abstract

Alzheimer’s disease (AD), an age-related progressive neurodegenerative disorder, exhibits reduced cognitive function with no cure to date. One of the reasons for AD is the accumulation of Amyloid-beta 42 (Aβ42) plaque(s) that trigger aberrant gene expression and signaling, which results in neuronal cell death by an unknown mechanism(s). Misexpression of human Aβ42 in the developing retina of *Drosophila* exhibits AD-like neuropathology. Small non-coding RNAs, microRNAs (miRNAs), post-transcriptionally regulate the expression of their target genes and thereby regulate different signaling pathways. In a forward genetic screen, we identified *miR-277* (human ortholog is hsa-miR-3660) as a genetic modifier of Aβ42-mediated neurodegeneration. Loss-of-function of *miR-277* enhances the Aβ42-mediated neurodegeneration. Whereas gain-of-function of *miR-277* in the *GMR* > *Aβ42* background downregulates cell death to maintain the number of neurons and thereby restores the retinal axonal targeting defects indicating the functional rescue. In addition, gain-of-function of *miR-277* rescues the eclosion- and climbing assays defects observed in *GMR* > *Aβ42* background. Thus, gain-of-function of *miR-277* rescues both structurally as well as functionally the Aβ42-mediated neurodegeneration. Furthermore, we identified *head involution defective* (*hid*), an evolutionarily conserved proapoptotic gene, as one of the targets of *miR-277* and validated these results using luciferase- and qPCR -assays. In the *GMR* > *Aβ42* background, the gain-of-function of *miR-277* results in the reduction of *hid* transcript levels to one-third of its levels as compared to *GMR* > *Aβ42* background alone. Here, we provide a novel molecular mechanism where *miR-277* targets and downregulates proapoptotic gene, *hid*, transcript levels to rescue Aβ42-mediated neurodegeneration by blocking cell death. These studies shed light on molecular mechanism(s) that mediate cell death response following Aβ42 accumulation seen in neurodegenerative disorders in humans and provide new therapeutic targets for neurodegeneration.

## Introduction

Alzheimer’s disease (AD), a fatal, progressive neurodegenerative disorder, is highly prevalent in the elderly aged 65 or above. It is characterized by progressive neuronal loss, cognitive decline, and memory defects [1, 2]. Furthermore, AD has been reported to be a leading cause of death worldwide with no available cure to date. The presence of amyloid plaques and intracellular tau neurofibrillary tangles are hallmarks of AD. Normally, the amyloid precursor protein (APP) is processed by α-secretase and γ-secretase to form amyloid-beta 40 (Aβ40) peptide. However, if APP is sequentially cleaved by β-secretase and γ-secretase, hydrophobic amyloid-beta 42 (Aβ42) peptides are formed. These Aβ42 monomers aggregate to form amyloid plaques and trigger the aberrant activation of signaling pathways and oxidative stress resulting in neuronal cell death [3–5]. Recently the FDA has approved lecanemab, an anti-amyloid antibody that slows down the cognitive and functional decline in early-stage AD patients, according to a phase 3 clinical trial [6]. Thus, amyloid plaques target(s) are being pursued as potential therapeutic targets [6].

Since the genetic machinery is conserved, several vertebrate and invertebrate model systems are being developed and used to study AD pathology and underlying mechanism(s) [7–11]. Invertebrate model system like *Drosophila melanogaster* can be easily genetically manipulated and offers a unique advantage to study the molecular mechanism(s) and pathogenesis of AD [11–14]. The *Drosophila* eye has been extensively used for modeling neurodegenerative models like AD [13, 15–17]. The adult compound eye, which develops from larval eye-antennal imaginal disc, is comprised of approximately 800 units called ommatidia. Each ommatidium consists of 8 photoreceptors and several support cells [17–21]. The undifferentiated epithelial cells in the eye imaginal disc begin to differentiate into retinal neurons and other cell types at the late larval stage onto pupal development [18, 19]. Between 24h and 40 h after pupa formation (APF), any extra cells are eliminated through programmed cell death (PCD) to refine the hexagonal lattice [18, 19]. The precise organization of *Drosophila* eye makes it highly sensitive to genetic manipulations and allows quick screening of large sample size [21–24]. Therefore, the *Drosophila* eye is employed to mimic many neurodegenerative disorders including AD and study the different signaling pathways as well as screen genetic modifiers and therapeutic targets for AD [13, 15, 25–27].

Using the Gal4/UAS transgenic target system [28], human Aβ42 is spatiotemporally misexpressed in the differentiating photoreceptor neurons of the developing eye to mimic AD [15–17]. This results in the accumulation of amyloid plaques exhibiting a progressive neurodegenerative phenotype in the *Drosophila* eye as compared to the wild-type eye, and thus phenocopies AD-like neuropathology [17, 26]. Therefore, the *Drosophila* eye model, due to repertoire of genetic tools, can be exploited for genome-wide screening to identify genetic modifiers of AD-like neuropathology. Accumulation of Aβ42 plaques triggers cell death, which is the primary cause of neurodegeneration in AD [29]. The proapoptotic factor *head involution defective* (*hid*) along with other caspases like Dronc, Drice, and Dark are involved in regulating PCD during pupal eye development [19]. In *Drosophila*, upon apoptotic stimuli, three proapoptotic genes: *head involution defective* (*hid*), *reaper* (*rpr*) and *grim (grim)* are expressed and thereby trigger cell death by inhibiting Drosophila inhibitor of apoptosis (DIAP1) [30–32]. Upon DIAP1 degradation, initiator caspase: Dronc (caspase 9) as well as effector caspase: Drice (caspase 3) are activated. This activation of the caspase cascade results in cell death. Caspase-dependent cell death can be prevented by high levels of baculovirus protein P35 [33]. Cell death observed in various human diseases is an outcome of aberrant signaling due to abnormal gene expression.

Complex gene regulatory mechanisms control the expression of genes during development. MicroRNAs (miRNAs) are small non-coding RNAs that post-transcriptionally regulate gene expression of different signaling pathways [34]. Recent findings indicate that miRNAs play a crucial role in modulating multiple signaling pathways associated with various diseases [35]. These miRNAs confer specificity to the RNA-induced silencing complex (RISC) through partial sequence complementarity with specific mRNA targets. Recruitment of miRNA and the RISC complex mostly results in repression of the target mRNA by an increase in turnover and/or translational inhibition. miRNAs regulate many biological events, including growth, development, differentiation, and neurodegenerative processes [36, 37]. Therefore, we hypothesized that miRNAs could regulate the expression of genes, which are members of signaling pathways that are involved in AD.

In a forward genetic screen using candidate miRNAs, we screened for potential genetic modifiers of Aβ42-mediated neurodegeneration phenotype. The rationale of the screen was to individually co-express a microRNA transgene along with *GMR* > *Aβ42* in the developing retina, and to screen for modifiers of the neurodegenerative phenotype. In this screen, we identified *miR-277* as a potential miRNAs genetic modifier using the *Drosophila* eye model. Here, we report that upregulation of *miR-277* rescues Aβ42-mediated neurodegeneration phenotype whereas downregulation of *miR-277* enhances Aβ42-mediated neurodegenerative phenotype of reduced eye with glazed surface. Furthermore, we have identified *hid* mRNA as a target of *miR-277* using bioinformatics, genetic and molecular approaches. Here, we present an insight on the underlying mechanism of how *miR-277* ameliorates Aβ42-mediated neurodegeneration by regulating proapoptotic gene, *hid*, transcript levels and demonstrate the novel neuroprotective role of *miR-277* in AD neuropathology.

## Materials and methods

### Stocks

The fly stocks used in this study are listed in Flybase (http://flybase.bio.indiana.edu). Stocks used in this study are *GMR-Gal4* [38], *Elav-Gal4* [39], *OK107-Gal4* [40, 41], *UAS-Aβ42* [42], *UAS miR-277*, *miR-277* mutant generated by TALEN editing, Df(3 R)*miR-277-34-*KO (BL#58908), *GMR-hid; GMR-Gal4*, *GMR*>*rpr*, *GMR*>*grim*, *hid*5’FWT-GFP [43]. The *UAS-Aβ42* transgenic flies were generated by microinjecting a UAS-construct where two tandem copies of human amyloid—β1-42 (Aβ42) fused to signal peptide for secretion were cloned [44, 45]. The rationale of bi-cistronic construct was to mimic APP duplications associated with early onset of familial AD and to express high levels of Aβ42 to induce strong eye phenotype.

### Generation of *miR-277* mutant by TALEN gene editing

We employed the transcription activator-like effector nucleases (TALEN) system for gene editing [46] to generate *miR-277* mutant lines. A 150 base pairs of flanking sequences that surrounded *miR-277* mature sequences were selected and added to the TALE-NT website (https://tale-nt.cac.cornell.edu/about) for designing target sites pairs for TALENs binding and editing [47, 48]. One pair of TALEN target sites, TALEN-*miR-277*-L_5′-GAAACTATCTGAAGCAT-3′ and TALEN-*miR-277*-R_5′-TCTGGAATGTCGTACC-3′, was chosen for the TALEN plasmids generation. The TALEN plasmids were synthesized by ZGENEBIO Biotech Inc. The midi-scale of TALEN plasmids was prepared by following the QIAGEN Plasmid Midi Kit (QIAGEN, #12145) and then transferred to BestGene Inc for *Drosophila* embryo injection services.

After embryo microinjection, all the G0 adults were crossed with stubble (TM3) balancer strains by combining one male and one female to generate the individual family. From each family, 15 G1 offspring were randomly picked up to establish the homozygous subfamily strains for mutant line screening. Genomic DNA isolations and PCR reactions were performed using the published protocol [49]. The primer pairs used for PCR reaction and Sanger sequencing are mentioned in Table 1.

**Table 1:** The list of primer pairs used for PCR reaction and Sanger sequencing.

Name	Sequence
TALEN_*miR-277* Screening F1	TTGGAGTTGCACCTTCGATTTCTGT
TALEN_*miR-277* Screening R1	TATACAGTTGAAAACTCTTCATAGA
TALEN_*miR-277* F1R1-PCR seq-F	AGCCATCCAGGAGATCGATA
TALEN_*miR-277* F1R1-PCR seq-R	CAGTGTCTTACAAACAAGTGG

Deshpande et al. [51].

### pTub-*miR-277* plasmid

A 500 base pairs of DNA fragment that included *miR-277* stem-loop precursor with each 200 base pairs of up and downstream flanking sequences were amplified from genomic DNA using the *miR-277* specific primer set (Table 2). The *miR-277* fragment was cloned into NotI/XhoI sites of the pTub-miR plasmid (gift from Dr. Stephen M. Cohen) to generate the pTub-*miR-277* plasmid.

**Table 2:** The list of primer pairs used to generate the pTub-miR-277 plasmid.

Name	Sequence
*miR-277*_NotI-F	GGCGCGGCCGCTTGGAGTTGCACCTTCGATTTCTGT
*miR-277*_XhoI-R	GGCCCTCGAGTATACAGTTGAAAACTCTTCATAGA

Deshpande et al. [51].

### Genetic crosses

Gal4/ UAS targeted misexpression system was used in our study [28]. The genetic crosses were maintained at 25 °C, while the egg-lays were transferred to 29 °C for further growth. Misexpression of Aβ42 in the differentiating retina (*GMR-Gal4* > *UAS-Aβ42*) exhibits a stronger neurodegenerative phenotype at 29 °C with no penetrance [38]. *GMR-Gal4* directs the expression of transgenes in the differentiating retinal precursor cells of the developing eye imaginal disc and pupal retina [38]. All crosses with Df(3 R)*miR-277*-*34*-KO (BL#58908) stock are mentioned as Df(3 R)*miR-277*KO.

### Adult eye imaging

The adult flies of similar age from both sexes were stored at −20 °C for approximately 2 h for imaging. After the incubation, the flies were mounted on a dissection needle. It was placed horizontally over a glass slide using clay putty. The adult eye was imaged on the Axiomager.Z1 Zeiss Apotome and the Z-stacks were obtained [42, 50]. The final images were generated by compiling individual stacks from the Z section using the extended depth of focus function of Axiovision software version 4.6.3.

### Frequency of eye phenotype

For each genetic cross, three independent sets of two hundred flies were screened (200 × 3 = 600) and the frequency of eye phenotype(s) was calculated [51]. The eye phenotypes were categorized as severely reduced eye, reduced eye, and rescue of neurodegenerative phenotype. Graphs were plotted in GraphPad Prism.

### Quantitative analyses of severity score of eye degenerative phenotype

We examined the eye phenotypes from 200 flies per genotype and these flies were selected for scoring according to the following criteria: “No-eye” was assigned to category 6, 80% eye degeneration was assigned to category 5, 60–80% eye degeneration was assigned to category 4, 40–60% eye degeneration was assigned to category 3, 20–40% eye degeneration was assigned to category 2, 0–20% eye degeneration was assigned to category 1 and wild-type was assigned to category 0 [51]. Comparisons were made using non-parametric: Mann–Whitney *t* test and graphs were plotted in GraphPad Prism.

### Quantitative analyses of relative surface area of the eye

The adult eye images were opened in Image J software, and region of interest (ROI) was drawn and represented as yellow dotted line. We measured the surface area of the eye of five flies per genotype by using Image J software and plotted graph in GraphPad Prism.

### Immunohistochemistry

Eye-antennal imaginal discs were dissected from the third instar larvae in cold 1× phosphate-buffered saline (PBS), fixed in 4% paraformaldehyde in 1× PBS for 20 min, and then quickly washed once in 1× PBS. It was followed by three washes in 1× PBST. The tissues were stained with a combination of antibodies following a previously published protocol [42, 52]. The primary antibodies used were rat anti-Embryonic Lethal Abnormal Vision (ELAV) (1:100; Developmental Studies Hybridoma Bank, DSHB), mouse anti-Discs-large (Dlg) (1:100; DSHB), and mouse anti-Chaoptin (24B10) (1:100; DSHB) [53]. Secondary antibodies (Jackson Laboratory) used were goat anti-rat IgG conjugated with Cy5 (1:250), and donkey anti-mouse IgG conjugated with Cy3 (1:250). The tissues were mounted in the antifading agent: Vectashield (Vector Laboratories). The immunofluorescent images were captured at 20× magnification by using Olympus Fluoview 3000 Laser Scanning Confocal Microscope [54]. All final figures were prepared using Adobe Photoshop software.

### Detection of cell death

Apoptosis was detected by using terminal deoxynucleotidyl transferase dUTP nick-end labeling (TUNEL) assay detection kit from Roche Diagnostics. TUNEL assay labels the DNA fragments in dying cells. This protocol involves labeling DNA breakage by adding fluorescently labeled nucleotides to free 3′-OH DNA ends in a template-independent manner using terminal deoxynucleotidyl transferase (TdT). The fluorescein labels (TMR red) incorporated in nucleotide polymers can be detected by fluorescence microscopy [32, 55]. After secondary-antibody staining, eye-antennal discs were blocked in 10% normal goat serum in phosphate-buffered saline with 0.2% Triton X-100 (PBST) and labeled for TUNEL assays using a cell-death detection kit from Roche Diagnostics. The TUNEL positive cells were counted from five sets of imaginal discs per genotype and were used for the statistical analysis using Microsoft Excel 2013 [56]. The graphs were plotted in GraphPad Prism, the *p* values were calculated using student’s *t* test, and the error bars represent standard error of mean (SEM). The symbols above the error bar signify *p value < 0.05, **p value < 0.01, ***p value < 0.001.

### Pupal retina staining

Early white pre-pupae per genotype were selected and kept on a moist kim-wipe in a petri plate. After 48 h of collection of pre-pupae, pupal retina was dissected in cold 1× PBS carefully from the pupa. The retinae were then fixed in 4% paraformaldehyde in 1× PBS for 20 min, and then washed once in 1× PBS. It was followed by three washes in 1× PBST. The dissection, washing, and antibody staining was done in nine well plate. The tissues were stained with a combination of antibodies following a previously published protocol [42, 52]. The primary antibodies used were rat anti-Embryonic Lethal Abnormal Vision (ELAV) (1:100; Developmental Studies Hybridoma Bank, DSHB), and mouse anti-Discs-large (Dlg) (1:100; DSHB). Secondary antibodies (Jackson Laboratory) used were goat anti-rat IgG conjugated with Cy5 (1:250), and donkey anti-mouse IgG conjugated with Cy3 (1:250). The tissues were mounted in Vectashield (Vector Laboratories). The immunofluorescent images were captured at ×60 magnification by using Olympus Fluoview 3000 Laser Scanning Confocal Microscope [54]. All final figures were prepared using Adobe Photoshop software. The pupal retina was opened in Image J and ROI of 500px × 500px was drawn and the number of photoreceptor cells and the number of pigment cells were counted within the ROI, respectively. The five sets of pupal retinae per genotype were used for statistical analysis using Microsoft Excel. The graphs were plotted in GraphPad Prism. The *p* values were calculated using student’s *t* test, and the error bars represent standard error of mean (SEM) *p value < 0.05, **p value < 0.01, ***p value < 0.001.

### DHE

The third instar larval eye-antennal imaginal discs were dissected in cold 1× Schneider’s *Drosophila* medium (Gibco, Cat. #21720024). The samples were incubated in Dihydroethidium (DHE, Life Technologies Cat. # D11347) dye solution [(1:300) in 1× PBS] [57, 58] for 5 min and were washed three times with cold 1× PBS. The eye discs were then mounted on a slide and were immediately imaged on a Laser Scanning Confocal microscope (Olympus Fluoview 3000) [54]. All final figures were prepared using Adobe Photoshop software. The number of ROS puncta were quantified from five sets of imaginal discs per genotype by using automated quantification method [58]. The Interactive H watershed plugin of Image J/Fiji free software was used for automated quantification and the statistical analysis was performed using Microsoft Excel [58]. The *p* values were calculated using student’s *t* test, and the error bars represent the standard error of mean (SEM) *p value < 0.05, **p value < 0.01, ***p value < 0.001.

### Luciferase reporter plasmid

The 3′UTRs of three apoptosis-related genes, *hid*, *dark,* and *drice*, were amplified from genomic DNA of W[1118] strain with the following gene-specific 3′UTR primer pairs (Table 3).

**Table 3:** The list of primer pairs used to generate three luciferase reporter plasmids.

Primer name	Sequence
*hid*_3′UTR-F	GCCGTGTAATTCTAGAAAGCGCAGGAGACGTGTAATCGAATGATCTAT
*hid*_3′UTR-R	AGGTCGACCTCGAGGCCTTTTACACATACACATAGATGTATTCATATA
*dark*_3′UTR-F	GCCGTGTAATTCTAGACGCCAGAAGTGCGTCCCTAGGCGG
*dark*_3′UTR-R	AGGTCGACCTCGAGGCCTGTGTTTTTCGTGAAATGTCGTTTATTTGGT
*drice*_3′UTR-F	GCCGTGTAATTCTAGATGGCTAATGGTATGGATCAAACGG
*drice*_3′UTR-R	AGGTCGACCTCGAGGCCTTGTTTTCAATCGGATTTATTAGCGGTTA

Deshpande et al. [51].

The 3′UTR DNA fragments were individually cloned into the XbaI/StuI sites of the pTub-ffluc reporter plasmid (gift from Dr. Stephen M. Cohen) to generate three luciferase reporter plasmids, pTub-ffuc-*hid*, pTub-ffuc-*dark* and pTub-ffuc-*drice*, by In-Fusion HD Cloning Kit (Clontech, 639646).

### Luciferase assay

The luciferase reporter gene assay has recently been adapted to test whether a certain mRNA is the target for a specific miRNA [59]. The day prior to transfection, *Drosophila* Schneider S2 (S2) cells were seeded at a density of 1–1.5 × 10^6^ cells per well in 12-well plate in Schneider’s *Drosophila* medium (SDM) (Gibco, Cat. #11720034) supplemented with 10% heat-inactivated fetal bovine serum (Gibco, Cat. #A3160402) and 1× penn/strep (Invitrogen, Cat. #15070-063). Before transfection, cells were washed with SDM only and changed the cultured medium into SDM (300 μl/well) only without serum and penn/strep for 1 h. The diluted plasmid reagent was prepared by mixing the following plasmids, 100 ng of pTub-rLuc (gift from Dr. Stephen M. Cohen), 100 ng of pTub-ffLuc luciferase reporter (containing 3′UTR sequence of the target gene), and 1 μg of pTub-*miR-277* with 100 μl SMD for each well. The diluted Cellfectin (Invitrogen, 10362-010) reagent was prepared by adding 5 μl Cellfectin in 100 μl SDM for each well and wait for 5 min. The diluted Cellfectin was mixed with diluted plasmids and waited for 30 min. The mixture was added to cells for 4–6 h transfection reaction, and then medium was changed into SDM with 10% FBS and 1× penn/strep. The pTub-*miR-1* (gift from Dr. Stephen M. Cohen) was performed at the same time in each transfection for negative control [60]. After 48-hour transfection, cells were lysed to detect the luciferase activities by using the Dual-Luciferase Reporter Assay System (Promega, E1910). Renilla luciferase activity provided normalization for firefly luciferase activity. The value of relative luciferase activities of the different pTub-ffLuc-3′UTR constructs was calculated from the normalization of the luciferase activities of *miR-1*. The targeting effect of *miR-277* on the luciferase gene expression can be shown as the relative luciferase activity.

### Real-time quantitative polymerase chain reaction

Real-time quantitative polymerase chain reaction (RT-qPCR) was performed according to the standardized protocol [61, 62]. Total RNA was extracted in 500 μl of TRIzol Reagent (Thermo Fisher, Cat. No. # 15596926) from twenty pairs of third instar larvae eye-antennal imaginal discs (*n* = 40), which were dissected from *GMR-Gal4, GMR* > *Aβ42, GMR>miR-277, GMR* > *Aβ42+ miR-277, GMR>miR-277* mutant*, GMR* > *Aβ42+ miR-277* mutant*, GMR* > Df(3 R)*miR-277*KO *and GMR* > *Aβ42* + Df(3 R)*miR-277*KO. The quality of isolated RNA was determined by using the Nanodrop 2000 spectrophotometer (Thermo Scientific). The quality of samples was checked by A260/A280 ratio which were >2. cDNA was produced from total RNA through RT-PCR using the first-strand cDNA synthesis kit (GE healthcare, Cat# 27926101). RT-qPCR was performed using iQ™ SYBR Green Supermix (Bio-Rad) and Bio-Rad iCycler (Bio-Rad) following the kit’s protocol for 25 μl. Primers used for *hid* were: (fwd: CCACCGACCAAGTGCTATAC; rev: CGGCGGATACTGGAAGATTT). Primers used for *miR-277* were: (fwd: GCGTGTCAGGAGTGCATTT; rev: GTACGTTCTGGAATGTCGTACC). The expression level of glyceraldehyde 3-phosphate dehydrogenase (GAPDH) (fwd: CAATGGATTTGGTCGCATCG; rev: CCGTTGACCACCAGG AAACC) was used as an internal control to normalize the results. The fold change was calculated relative to the expression level of the respective control using delta deltaCT (ΔΔCT) method [61].

### Eclosion assay

Eclosion assays are used for screening the effects of various genetic backgrounds on eclosion of flies. We used *Elav-Gal4* line to drive the expression of *Aβ42* and other transgene in the central nervous system [39]. We collected eggs on a grape plate from *Elav-Gal4* (control), *Elav* > *Aβ42*, *Elav* > *Aβ42* *+ miR-277*, *Elav* > *Aβ42* + *miR-277* mutant and *Elav* > *Aβ42* + Df(3 R)*miR-277*KO. We seeded the first instar larvae (30 in each set) from a synchronous culture in each vial. 270 larvae (9 sets of 30 larvae) were counted for each cross. The larvae were allowed to develop to adulthood, and the eclosion rate [42] was counted. All unhatched pupae were also counted. The graph was plotted in GraphPad Prism.

### Climbing assay

Climbing assays were performed to characterize the locomotor dysfunction. We used *OK107-Gal4* line to drive the expression of *Aβ42* and other transgene in the larval mushroom body as well as in adult mushroom body lobes [40, 41]. Since, *Elav* > *Aβ42* flies were lethal, we used *OK* > *Aβ42* flies for detecting if *miR-277* can affect the locomotor defects observed in AD flies. Flies were aged from 1 to 30 days in the regular food. Groups of 10 flies per genotype were transferred into cylindrical glass tube after anesthetization and left for 5-10 min for the revival and acclimatization at room temperature. Tubes were marked at 10 cm above the bottom of the vial. After acclimatization, the flies were gently tapped down to the bottom of vial, and the number of flies that crossed the 10 cm mark were recorded after 10 sec. Three trials were performed, and numbers were then averaged, and the resulting mean was used as the overall value for each single group of flies. For all genotypes, three replicates were carried out.

## Results

### *miR-277* is a genetic modifier of Aβ42-mediated neurodegeneration

**Fig. 1:**
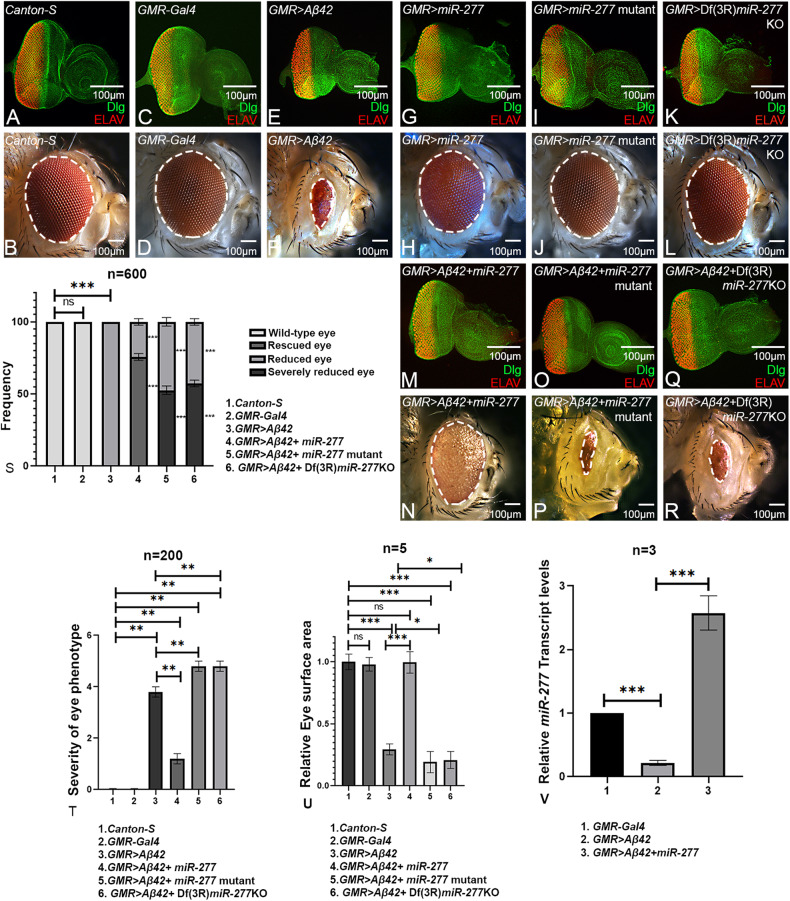
Gain-of-function of *miR-277* modulates the Aβ42-mediated neurodegeneration. **A** Wild-type larval eye imaginal disc develops in to **B** an adult eye comprises of ~800 organized ommatidia. Note that the eye imaginal disc is stained with a membrane-specific marker, discs large (Dlg; green), and a pan neural marker ELAV (red) that marks the photoreceptors (**C**, **D**) *GMR-Gal4*, drives the expression of target transgenes in the developing eye, serves as a control. **E**, **F** Misexpression of human Aβ42 in the developing (**E**) eye imaginal disc (*GMR* > *Aβ42*) leads to highly reduced (**F**) adult eye phenotype with fused ommatidia. **G**, **H** *GMR>miR-277,* **I**, **J** *GMR>miR-277* mutant, and **K**, **L** *GMR* > Df(3 R)*miR-277*KO eye imaginal discs and adult eye also served as controls. **M**, **N** Gain-of-function of *miR-277* in the background of *GMR* > *Aβ42* (*GMR* > *Aβ42+ miR-277*) results in significant rescue in eye disc and adult eye as compared to the *GMR* > *Aβ42*. **O**–**R** However, reducing *miR-277* levels in *GMR* > *Aβ42* flies using (**O**, **P**) *miR-277* mutant (*GMR* > *Aβ42+ miR-277* mutant), (**Q**, **R**) Df(3 R)*miR-277*KO [*GMR* > *Aβ42* + Df(3 R)*miR-277*KO] enhances *GMR* > *Aβ42* neurodegenerative phenotype. **S** Bar graph shows frequency as average between 3 repetitions. Two hundred flies were counted per repetition (200 × 3 = 600) to calculate the frequency for each genotype (1. *GMR-Gal4*, 2. *GMR* > *Aβ42*, 3. *GMR* > *Aβ42+miR-277*, 4. *GMR* > *Aβ42+miR-277* mutant, 5. *GMR* > *Aβ42* + Df(3 R)*miR-277*KO*)*. Statistical analysis was performed using the student’s *t* test for independent samples. **T** Quantitative analyses of severity score of neurodegenerative phenotypes*(*s) in eye. Flies from each genotype were selected for scoring according to the criteria described in methods section. Comparisons were made using non-Parametric: Mann–Whitney *t* test. **U** Quantitative analyses of area of the eye. The surface area of the eye (within white dotted line) was calculated using Image J. Statistical analysis was performed using the student’s *t* test for independent samples. The surface area is significantly rescued in *GMR* > *Aβ42+miR-277* (*n* = 5; *p* = 0.0000000146) whereas significantly reduced in *GMR* > *Aβ42+miR-277* mutant (*n* = 5; *p* = 0.017) and *GMR* > *Aβ42* + Df(3 R)*miR-277*KO (*n* = 5; *p* = 0.02) as compared to *GMR* > *Aβ42*. **V** Relative expression of *miR-277* at the transcriptional level using quantitative PCR (qPCR) in genotypes (1. *GMR-Gal4* 2. *GMR* > *Aβ42*, 3. *GMR* > *Aβ42+ miR-277*). Triplicate was used for the calculation. Statistical analysis was performed using student’s *t* test for independent samples. The data plotted shows mean ± SEM (Standard Error of Mean), and symbols above the error bar signify as ***p value < 0.001, **p value < 0.01, *p value < 0.05, and not significant (ns), *p* value > 0.05 respectively. The orientation of all imaginal discs is identical with posterior to the left and dorsal up. Scale bar = 100 μm.

*Drosophila* larval eye imaginal disc (Fig. 1A) develops into an adult compound eye comprising around 800-unit eyes called ommatidia (Fig. 1B). Note that the eye imaginal disc is stained for membrane-specific marker- discs large (Dlg, green) and pan neural marker- embryonic lethal abnormal vision (Elav, red) (Fig. 1A, C, E, G, I, K, M, O, Q). The *GMR-Gal4* driver flies have wild-type adult eye (Fig. 1D) and eye imaginal discs (Fig. 1C). Misexpression of human Aβ42 in the differentiating retinal neurons of the developing eye by using *GMR-Gal4* driver (*GMR* > *Aβ42*), results in a progressive neurodegenerative phenotype with increased spaces in the photoreceptors mostly at the posterior margin of the eye disc (Fig. 1E), which further worsens in the adult eye as evident from the reduced eye size, disorganized and fused ommatidia in the adult eye (Fig. 1F). The progressive neurodegenerative phenotype of highly reduced adult eye has 100% penetrance (*n* = 600, 600/600 = 100%) (Fig. 1F, S, T, U) [17]. In a forward genetic screen (Supplementary Figure 1), we identified *miRNA−277* (*miR-277*) as the modifier of the neurodegenerative phenotype of *GMR* > *Aβ42* (Fig. 1M, N, S, T, U). Misexpression of *miR-277* alone (*GMR* *>* *miR-277)* serves as a control and exhibits near-normal eye phenotype in the eye imaginal disc and adult eye (Fig. 1G, H). Whereas gain-of-function of *miR-277* in *GMR* > *Aβ42* (*GMR* > *Aβ42*+*miR-277*) background (Fig. 1M, N, S, T, U) significantly rescues the Aβ42-mediated neurodegeneration (Fig. 1E, F, S, T, U) as seen in eye imaginal disc as well as the adult eye (*n* = 600, 600/600 = 100%).

To further validate our hypothesis, we investigated the loss-of-function of *miR-277* by using *miR-277* mutant (Fig. 1I, J) generated by TALEN system (Supplementary Figure 2A–C) or a deficiency, Df(3 R) *miR-277*KO that knocks out *miR-277* function [*GMR* > Df(3 R)*miR-277*KO; Fig. 1K, L] in *GMR-Gal4* background. Interestingly, loss-of-function of *miR-277* in *GMR* > *Aβ42* background (*GMR* > *Aβ42+ miR-277* mutant) enhances the neuronal loss and hence show reduced eye phenotype (Fig. 1O, P, S, T, U) (Supplementary Figure 2) as compared to the *GMR* > *Aβ42* alone (Fig. 1F). The severity of the phenotype is more in frequency with Df(3 R)*miR-277*KO (*GMR* > *Aβ42* + Df(3 R)*miR-277*KO) (*n* = 600, 333/600 = 55.5%) (Fig. 1S) than with the molecular mutant (*n* = 600, 306/600 = 51%) (Fig. 1S). Moreover, gain-of-function of *miR-277* in the *GMR* > *Aβ42* flies significantly suppressed the neurodegenerative phenotype (Fig. 1T) and increased the surface area of the eye (Fig. 1U). This further validates our findings from the forward genetic screen that *miR-277* is a genetic modifier of Aβ42-mediated neurodegeneration in the *Drosophila* eye.

We further quantitated the *miR-277* transcript levels by qPCR and found that *miR-277* levels were reduced to half in *GMR* > *Aβ42* background as compared to the control, *GMR-Gal4*. Furthermore, *miR-277* levels were increased by 2.5 folds in *GMR* > *Aβ42+ miR-277* background as compared to the control (Fig. 1V). Thus, our data suggest that a reduction in *miR-277* levels triggers neurodegenerative response.

### Gain-of-function of *miR-277* can restore axonal targeting defects

**Fig. 2:**
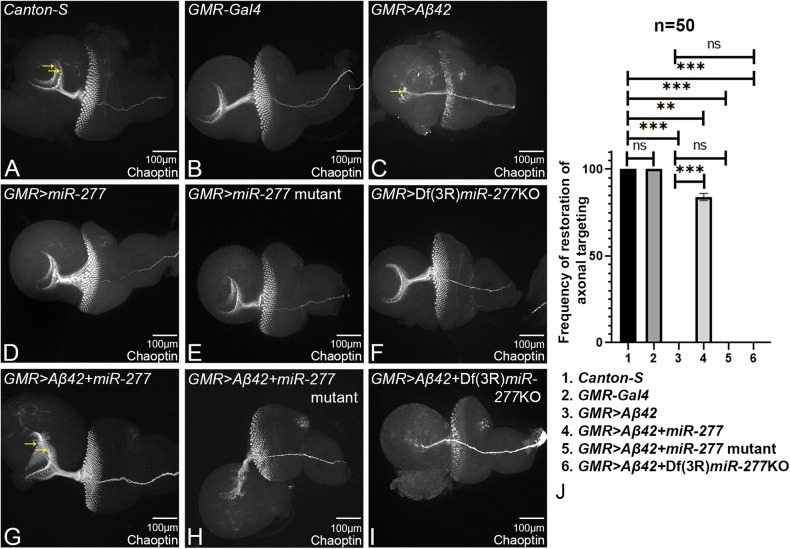
Gain-of-function of *miR-277* can restore axonal targeting impaired by Aβ42-mediated neurodegeneration. Chaoptin (24B10) marks the photoreceptors and their neurons. The photoreceptor neurons bundle up in the optic stalk and innervate the medulla and lamina of the brain. **A** In the wild-type eye imaginal disc, the retinal axons marked by 24B10 innervates the two layers of the brain marked with yellow arrows. **B** *GMR-Gal4* serves as the control. **C** Misexpression of Aβ42 (*GMR* > *Aβ42*) results in impaired axonal targeting from retina to the brain. The retinal axons fail to innervate the two layers of the brain and end abruptly. Controls (**D**) *GMR>miR-277* (**E**) *GMR>miR-277* mutant, and (**F**) *GMR* > Df(3 R)*miR-277*KO do not impact axonal targeting. **G** Gain-of-function of *miR-277* in the background of *GMR* > *Aβ42* (*GMR* > *Aβ42+ miR-277*) significantly restores the axonal targeting to near wild*-*type. Loss-of-function of *miR-277* in *GMR* > *Aβ42* flies using (**H**) *miR-277* mutant (*GMR* > *Aβ42+ miR-277* mutant), and (**I**) Df(3 R)*miR-277*KO (*GMR* > *Aβ42* + Df(3 R)*miR-277*KO) disrupt the axonal targeting. The orientation of all imaginal discs is identical with posterior to the left and dorsal up. The magnification of all eye imaginal discs is ×20. **J** Bar graph shows the frequency of restoration of axonal targeting phenotype. Sample size was 5 for each genotype 1. *GMR-Gal4*, 2. *GMR* > *Aβ42*, 3. *GMR* > *Aβ42+miR-277*, 4. G*MR* > *Aβ42+miR-277* mutant, 5. *GMR* > *Aβ42* + Df(3 R)*miR-277*KO. *GMR* > *Aβ42+miR-277* significantly restores the axonal targeting (*n* = 5; *p* = 0.000029) whereas *GMR* > *Aβ42+ miR-277* mutant (*n* = 5; *p* = 0.37) and *GMR* > *Aβ42* + Df(3 R)*miR-277*KO (*n* = 5; *p* = 0.38) disrupt the axonal targeting as compared to *GMR* > *Aβ42*. The data plotted shows mean ± SEM (Standard Error of Mean), and symbols above the error bar signify as ***p value < 0.001, **p value < 0.01, *p value < 0.05, and not significant (ns), *p* value > 0.05 respectively. Scale bar = 100 μm.

In AD, neurons die due to the deposition of amyloid plaques. Since *miR-277* can rescue the reduced eye phenotype of *GMR* > *Aβ42*, we investigated if *miR-277* can rescue the functionality of the neurons. One of the facets of the neurodegenerative phenotypes in retinal neurons is the disruption of axonal transport due to impaired axonal targeting and guidance from retinal neurons to the brain. We used Chaoptin (24B10, DSHB), a reliable marker for axonal targeting that marks the photoreceptor neurons and their axons [53]. During the late third instar eye imaginal disc, R1-R6 axons of each ommatidium project to the lamina, whereas R7 and R8 axons project to the medulla, a separate layer of the optic lobes [16, 63]. We counted a total of *n* = 50 eye discs per genotype, and frequency of eye discs indicating rescue of axonal targeting from *GMR* > *Aβ42* was recorded (Fig. 2A–J). The wild-type (*n* = 50) 100% (Fig. 2A, J) and *GMR-Gal4* (*n* = 50) 100% (Fig. 2B, J) eye imaginal discs showed similar axonal projections in the brain. In comparison to the controls, *GMR* > *Aβ42* imaginal discs exhibit severe defects in axonal targeting, which contributes to the neurodegenerative phenotype in AD (Fig. 2C, J) [16, 64]. Misexpression of *miR-277* in *GMR* > *Aβ42* (*GMR* > *Aβ42+ miR-277*; Fig. 2G, J) background, significantly restored the axonal targeting in comparison to the *GMR* > *Aβ42* (Fig. 2C, J). However, *GMR> miR-277* (Fig. 2D), or *GMR>miR-277* mutant (Fig. 2E), or *GMR* > Df(3 R)*miR-277*KO (Fig. 2F) alone did not show any axonal targeting defects. The axonal targeting was further impaired or worsened when *miR-277* levels were downregulated in the *GMR* > *Aβ42* background by either *miR-277* mutant (*GMR* > *Aβ42 + miR277* mutant, Fig. 2H, J) or by Df(3 R)*miR-277*KO (*GMR* > *Aβ42* + Df(3 R)*miR-277*KO, Fig. 2I, J). This evidence suggests that upregulating the levels of *miR-277* restores the axonal targeting defects, as seen in the *GMR* > *Aβ42* background. It has been shown that impaired axonal targeting is associated with neuronal cell death. Therefore, we tested the effects of modulation of *miR-277* in cell death in *GMR* > *Aβ42* background.

### Gain-of-function of *miR-277* can block Aβ42-mediated cell death

**Fig. 3:**
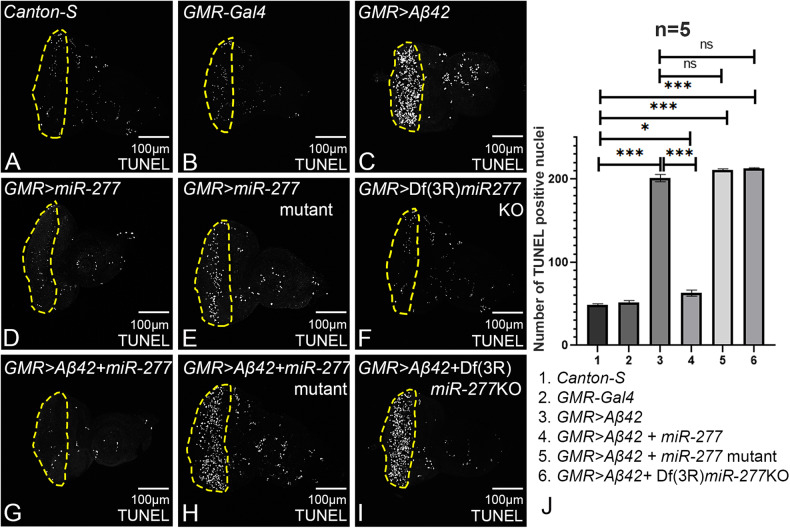
Gain-of-function of *miR-277* in the *GMR* > *Aβ42* background can prevent neuronal cell death. Terminal deoxynucleotidyl transferase-mediated dUTP nick-end labeling (TUNEL) assay is employed to mark the cells undergoing cell death. **A** Wild-type (*Canton-S*) and **B** *GMR-Gal4* eye imaginal discs show a few TUNEL-positive nuclei. **C** Misexpression of Aβ42 using *GMR-Gal4* driver (*GMR* > *Aβ42*) shows increased levels of TUNEL positive nuclei as compared to (**A**) wild-type eye imaginal disc. **D** *GMR>miR-277,* **E** *GMR>miR-277* mutant, and **F** *GMR* > Df(3 R)*miR-277*KO serve as controls. **G** Gain-of-function of *miR-277* in the background of *GMR* > *Aβ42* (*GMR* > *Aβ42+ miR-277*) significantly rescue the cell death as compared to *GMR* > *Aβ42*. Loss-of-function of *miR-277* in *GMR* > *Aβ42* flies using **H** *miR-277* mutant (*GMR* > *Aβ42+ miR-277* mutant), and (**I**) Df(3 R)*miR-277*KO (*GMR* > *Aβ42* + Df(3 R)*miR-277*KO) show elevated levels of TUNEL positive nuclei. **A**–**I** TUNEL-positive nuclei were counted within yellow dotted line, the region of interest, for the statistical analysis. **A**, **C**, **G**–**J** TUNEL-positive nuclei in photoreceptor cells of five eye imaginal discs per genotype were counted (1. *Canton-S*, 2. *GMR-Gal4, 3. GMR* > *Aβ42*, 4. *GMR>miR-277*, 5*. GMR* > *Aβ42+ miR-277*, 6.*GMR> miR-277* mutant, 7. *GMR* > *Aβ42+ miR-277* mutant, 8. *GMR* > Df(3 R)*miR-277*KO, 9*. GMR* > *Aβ42* + Df(3 R)*miR-277*KO). Statistical analysis was performed using student’s *t* test for independent samples. *GMR* > *Aβ42+miR-277* exhibits significant reduction in TUNEL-positive nuclei as compared to *GMR* > *Aβ42* (*n* = 5; *p* = 0.000000014) whereas *GMR* > *Aβ42+ miR-277* mutant (*n* = 5; *p* = 0.083) and *GMR* > *Aβ42* + Df(3 R)*miR-277*KO (*n* = 5; *p* = 0.056) show slight increase in TUNEL positive nuclei as compared to *GMR* > *Aβ42*. The data plotted shows mean ± SEM (Standard Error of Mean), and symbols above the error bar signify as ***p value < 0.001, **p value < 0.01, *p value < 0.05, and not significant (ns), *p* value > 0.05 respectively. The orientation of all imaginal discs is identical with posterior to the left and dorsal up. Scale bar = 100 μm.

We employed terminal deoxynucleotidyl transferase dUTP nick-end labeling (TUNEL) staining to mark the nuclei of dying cells. Fluorescein-dUTP is transferred by the enzyme terminal deoxynucleotides transferase (TdT) at 3’OH where single or double-strand breaks occur during apoptosis [55, 56]. The TUNEL-positive cells were counted within yellow dotted ROI from five imaginal discs of each genotype and were used for statistical analysis. A few cells undergo cell death in the wild-type eye imaginal disc (Fig. 3A, J), whereas the *GMR* > *Aβ42* eye imaginal disc shows significantly increased TUNEL positive nuclei (Fig. 3C, J). Gain-of-function of *miR-277* in the *GMR* > *Aβ42* background (*GMR* > *Aβ42+miR-277*; Fig. 3G, J) results in a significant reduction (~4 times) in the number of dying cells as compared to the *GMR* > *Aβ42* (Fig. 3C, J). Loss-of-function of *miR-277* in *GMR* > *Aβ42* background either by *miR-277* mutant (*GMR* > *Aβ42+ miR-277* mutant; Fig. 3H, J) or by Df(3 R)*miR-277*KO (*GMR* > *Aβ42* + Df(3 R)*miR-277*KO; Fig. 3I, J), results in significant increase in number of dying nuclei as compared to the wild-type (Fig. 3A, J). There is a slight increase in TUNEL-positive nuclei in case of deficiency of *miR-277* (*GMR* > *Aβ42* + Df(3 R)*miR-277*KO; Fig. 3I, J) as compared to the *miR-277* mutant (*GMR* > *Aβ42+ miR-277* mutant; Fig. 3H, J). Hence, TUNEL data suggests that the *miR-277* might downregulate cell death caused due to Aβ42-mediated neurodegeneration.

**Fig. 4:**
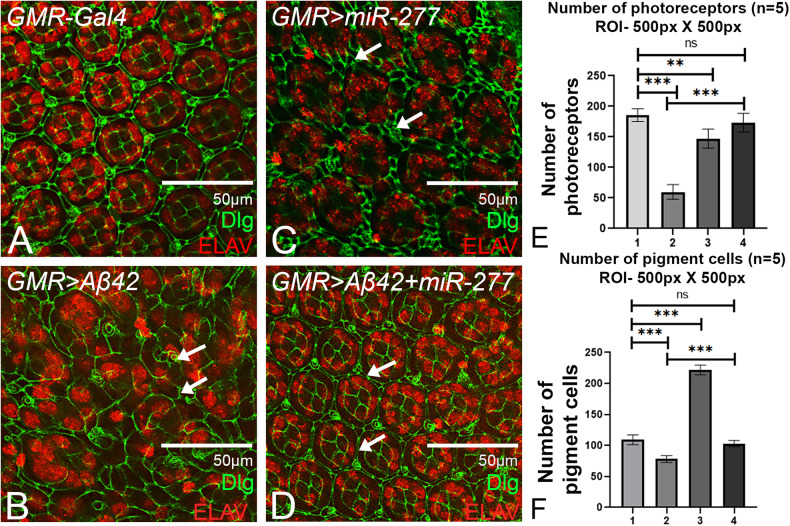
Gain-of-function of *miR-277* downregulates cell death and plays a role in growth. Pupal retinae were stained with Discs large (Dlg) which marks the membrane (green), and a pan neural marker ELAV (red) which marks photoreceptors. **A** Pupal retina of wild-type shows hexagonal ommatidia and single layer of interommatidial cells. **B** *GMR* > *Aβ42* shows disrupted pupal retina as compared to **A** wild-type pupal retina. **C** pupal retina of *GMR>miR-277* shows excess interommatidial cells as compared to the control (**A**) wild-type pupal retina. **D** *GMR* > *Aβ42+ miR-277* restores the number and shape of ommatidial and interommatidial cells. **E** Bar graph represents the number of photoreceptors within the area of 500px × 500px. We examined five pupal retinae per genotype (*n* = 5) (1. *GMR-Gal4*, 2. *GMR* > *Aβ42*, 3. *GMR>miR-277*, 4. *GMR* > *Aβ42+ miR-277*). Statistical analysis was performed using student’s *t* test for independent samples. The number of photoreceptors is significantly restored in *GMR* > *Aβ42+ miR-277* (*n* = 5; *p* = 0.0000018) as compared to *GMR* > *Aβ42*. **F** Bar graph represents the number of pigment cells (secondary, tertiary, and bristle cells) within the area of 500px × 500px. We examined five pupal retinae per genotype (*n* = 5) (1. *GMR-Gal4*, 2. *GMR* > *Aβ42*, 3. *GMR>miR-277*, 4. *GMR* > *Aβ42+ miR-277*). Statistical analysis was performed using student’s *t* test for independent samples. The number of pigment cells is significantly restored in *GMR* > *Aβ42+ miR-277* (*n* = 5; *p* = 0.000064) as compared to *GMR* > *Aβ42*. Error bars show standard error of mean (mean ± SEM), and symbols above the error bar signify as ***p value < 0.001, **p value < 0.01, * *p* value < 0.05, and not significant (ns), *p* value > 0.05 respectively. Scale bar = 50 μm.

To investigate further if *miR-277* can inhibit apoptosis during later stages of development, we performed pupal retina staining at 48 h after pupa formation (APF). During late larval development, the photoreceptor and cone cells are determined, following which the interommatidial cells (IOC’s) are specified to become pigment cells. Interommatidial (or pigment cells) surround the centrally located photoreceptor and cone cells, generating a precise, repeating hexagonal structure (ommatidium), which can be visualized during the pupal stage. This hexagonal lattice is formed due to programmed cell death (PCD), which occurs during 24–40 h APF to eliminate extra interommatidial cells [18, 65]. We dissected the pupal retina at 48 h APF, when PCD was over. The number of photoreceptor nuclei and pigment cells from five individual areas was selected from five different pupal retinae for each genotype for analysis. The control *GMR-Gal4* shows hexagonal arrangement of ommatidia and a monolayer of secondary and tertiary pigment cells (Fig. 4A, E, F). In contrast, the basic ommatidial organization is disrupted in *GMR* > *Aβ42* pupal retina due to cell death, which results in the fusion of photoreceptors among neighboring ommatidia as evident from ELAV staining that marks the photoreceptor nuclei (Fig. 4B, E). In addition, the membrane of primary and secondary pigment cells is lost, as marked by Dlg (Fig. 4B, F). We observed multiple layers of secondary and tertiary pigment cells resulting in extra pigment cells when *miR-277* was misexpressed in the GMR domain (*GMR>miR-277*; Fig. 4C, F). Since *miR-277* is an anti-apoptotic miRNA, in *GMR>miR-27*7 background, extra IOC’s accumulate between the ommatidia of the pupal retina as programmed cell death is inhibited. Interestingly the disruption of cell membrane, number of pigment cells, and fused ommatidia were significantly restored to near wild-type when *miR-277* was misexpressed in the *GMR* > *Aβ42* background (*GMR* > *Aβ42+miR-277*; Fig. 4D–F). Therefore, these results further validated our hypothesis that higher levels of *miR-277* suppress the neurodegenerative phenotype of Aβ42 aggregate accumulation. Hence, *miR-277* could be an anti-apoptotic miRNA. It has been previously reported that Aβ42 aggregate triggers the production of reactive oxygen species (ROS) [58, 66, 67].

### Gain-of-function of *miR-277* downregulates ROS production

**Fig. 5:**
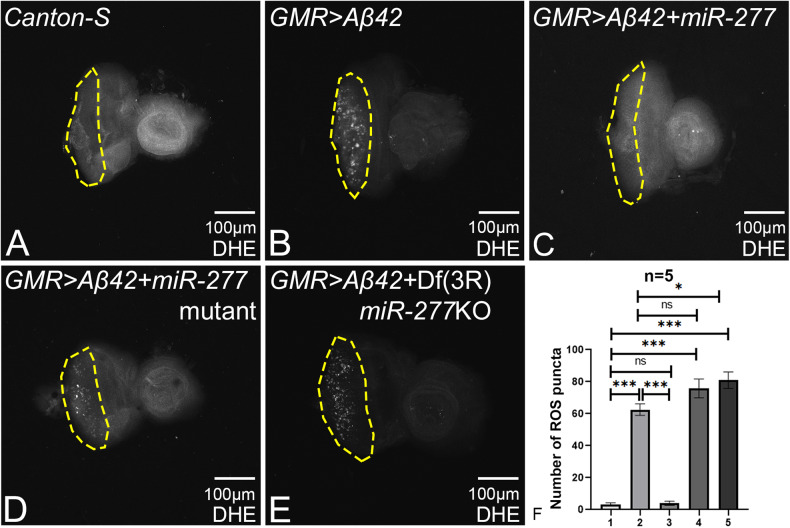
Modulation of *miR-277* in GMR > Aβ42 flies reduces ROS production. Dihydroethidium (DHE) is employed to detect ROS production in cells. **A** Wild-type (*Canton-S*) eye imaginal discs show a few ROS puncta. **B** *GMR* > *Aβ42* shows elevated ROS puncta as compared to **A** wild-type eye imaginal disc. **C** Gain-of-function of *miR-277* in the background of *GMR* > *Aβ42* (*GMR* > *Aβ42+ miR-277*) significantly reduced the ROS production as compared to *GMR* > *Aβ42*. Loss-of-function of *miR-277* in *GMR* > *Aβ42* background using **D** *miR-277* mutant (*GMR* > *Aβ42+ miR-277* mutant), and **E** Df(3 R) *miR-277*KO [*GMR* > *Aβ42* + Df(3 R)*miR-277*KO] result in increased ROS production. **A**–**F** ROS puncta were counted within yellow dotted line, the region of interest, for the statistical analysis. ROS puncta were quantified in photoreceptor cells of five eye imaginal discs per genotype (1. *Canton-S*, 2. *GMR* > *Aβ42*, 3. *GMR* > *Aβ42+ miR-277*, 4. *GMR* > *Aβ42+ miR-277* mutant, 5. *GMR* > *Aβ42* + Df(3 R)*miR-277*KO). Statistical analysis was performed using student’s *t* test for independent samples*. GMR* > *Aβ42+miR-277* exhibits significant reduction in ROS puncta as compared to *GMR* > *Aβ42* (*n* = 5; *p* = 0.000024), whereas *GMR* > *Aβ42+ miR-277* mutant (*n* = 5; *p* = 0.1) shows slight increase and *GMR* > *Aβ42* + Df(3 R)*miR-277*KO (*n* = 5; *p* = 0.02) shows significant increase in ROS puncta as compared to *GMR* > *Aβ42*. The data plotted shows mean ± SEM (Standard Error of Mean), and symbols above the error bar signify as ***p value < 0.001, **p value < 0.01, *p value < 0.05, and not significant (ns), *p* value > 0.05, respectively. The orientation of all imaginal discs is identical with posterior to the left and dorsal up. Scale bar = 100 μm.

The accumulation of amyloid plaques triggers oxidative stress in the neurons resulting in an imbalance in the generation of reactive oxygen species (ROS) and antioxidant defense mechanism [17, 58, 68, 69]. Excessive generation of ROS levels leads to oxidative modification of biomolecules in postmitotic neurons that are associated with AD pathology [58, 70, 71]. Hence, we measured ROS levels using dihydroethidium (DHE) staining in eye-antennal imaginal discs when *miR-277* levels were modulated in the background of *GMR* > *Aβ42* flies. DHE is oxidized by superoxide radical to form 2-hydroxyethidium, which intercalates with DNA and provides signal at 550 nm in cells where ROS is produced [58, 72, 73]. The ROS puncta were calculated (within the yellow dotted line that marks ROI) from five imaginal discs per genotype and were used for statistical analysis (Fig. 5A–F). Misexpression of Aβ42 (*GMR* > *Aβ42*; Fig. 5B, F) results in prominent ROS production as compared to minimal ROS levels in wild-type control *Canton-S* (Fig. 5A, F). Interestingly, gain-of-function of *miR-277* in the *GMR* > *Aβ42* background (*GMR* > *Aβ42 + miR-277*; Fig. 5C, F) shows reduced or similar levels of ROS signal as compared to the wild-type (Fig. 5A, F). Loss-of-function of *miR-277* in the background of *GMR* > *Aβ42* either by *miR-277* mutant (*GMR* > *Aβ42 + miR-277* mutant; Fig. 5D, F) and Df(3 R)*miR-277*KO (*GMR* > *Aβ42* + Df(3 R)*miR-277*KO; Fig. 5E, F) show increased ROS levels as compared to the *GMR* > *Aβ42* (Fig. 5B, F). Hence, high levels of *miR-277* can downregulate ROS levels in *GMR* > *Aβ42* flies.

### Gain-of-function of *miR-277* enhances the eclosion rate of Aβ42 expressing flies

**Fig. 6:**
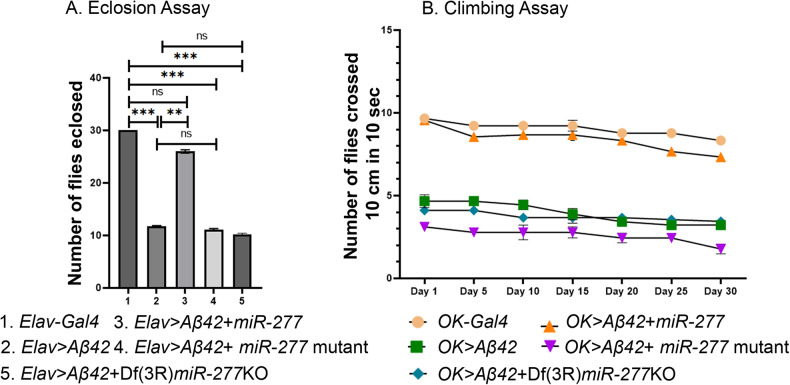
Overexpression of *miR-277* reduces the mortality rate and rescues the climbing defects in AD flies. **A** The bar graph represents the number of flies eclosed. We compared the number of flies eclosed in 1. *Elav Gal4*, 2. *Elav* > *Aβ42*, 3. *Elav* > *Aβ42*+ *miR-277* and 4. *Elav* > *Aβ42+ miR-277* mutant 5. *Elav* > *Aβ42* + Df^*(*^3 R)*miR-277*KO background and validated that overexpression of *miR-277* in the *Elav* > *Aβ42* background rescues the *Elav* > *Aβ42* mortality rate. We counted 270 flies in three independent biological sets from each background and plotted it on a graph in GraphPad Prism. The data plotted shows mean ± SEM (Standard Error of Mean), and symbols above the error bar signify as ***p value < 0.001, **p value < 0.01, *p value < 0.05, and not significant (ns), *p* value > 0.05, respectively. **B** Improved climbing activity when *miR-277* is overexpressed in AD flies. We compared the number of flies which crossed 10 cm from the bottom of the vial. Graph represents the climbing activity of the flies from day 1–day 30.

Since overexpression of *miR-277* rescues the neurodegeneration phenotype of flies expressing *Aβ42*, we wanted to check if *miR-277* can rescue the defects in eclosion rate observed in AD flies. To address this, we overexpressed *miR-277* in the central nervous system by using *Elav>Gal4* driver [42]. All wild-type flies that serve as control did not show any lethality and had 100% eclosion rate (Fig. 6A, *n* = 270, 100%). Conversely, a high mortality rate was observed in *Elav* > *Aβ42* as only 37% (*n* = 270) of the flies could hatch out and survive whereas the remaining 63% population failed to eclose as adults. However, overexpression of *miR-277* resulted in significant improvement in the eclosion rate of *Elav* > *Aβ42* flies (*Elav* > *Aβ42+ miR-277*; Fig. 6A, *n* = 270) as 86.6% of flies hatched. Whereas when *miR-277* was downregulated using *miR-277* mutant (*Elav* > *Aβ42+ miR-277* mutant) or Df(3 R)*miR-277*KO deficiency *(Elav* > *Aβ42* + Df(3 R)*miR-277*KO*),* the eclosion rates reduced to 35.3% and 30% respectively (Fig. 6A, *n* = 270). Thus, there is a significant improvement in eclosion rates when *miR-277* is overexpressed in *Elav* > *Aβ42* background.

### *miR-277* rescues locomotor defects in AD flies

We assessed the locomotor function of AD flies in the *miR-277* gain-of-function background using the climbing assay. Since we observed lethality with *Elav* > *Aβ42* flies, we used OK107 Gal4 to drive human Aβ42 misexpression in the mushroom body neurons and their axons [51, 74–76]. Previous studies have shown that OK107 Gal4 can be used to assess the locomotion [74–76]. First, we overexpressed Aβ42 in mushroom body of larvae and adult using *OK-Gal4* driver line and assayed the locomotor dysfunction in AD flies [40, 41]. We then compared the locomotor dysfunction of *OK* > *Aβ42* flies with the ones where the levels of *miR-277* expression were modulated in *OK* > *Aβ42* background. We checked their climbing ability from day 1 to day 30 of their eclosion as AD is an age-dependent disorder. The flies were cultured from the embryonic stage to adult stage on regular food. We performed climbing assay of one-day-old to one-month-old flies. We found climbing defects in *OK* > *Aβ42* flies on day 1 and further worsened through day 30 as compared to *OK-Gal4* (Fig. 6B). Overexpression of *miR-277* in *OK* > *Aβ42* (*OK* > *Aβ42+ miR-277*) flies exhibit significant rescue in climbing ability as compared to the control *OK* > *Aβ42* flies (Fig. 6B). The climbing activity of *OK* > *Aβ42+ miR-277* slightly decreased from day 20 to day 30 due to aging (Fig. 6B). Conversely, downregulation of *miR-277* in *OK* > *Aβ42* using *miR-277* mutant (*OK* > *Aβ42+ miR-277* mutant) or Df(3 R)*miR-277*KO (*OK* > *Aβ42* + Df(3 R)*miR-277*KO) flies showed severe climbing defects as compared to *OK* > *Aβ42* control flies which progressively worsened with the age (Fig. 6B). This data further demonstrates that *miR-277* overexpression can restore the climbing defects of the *OK* > *Aβ42* flies.

### *hid* is one of the targets of *miR-277*

To discern the molecular mechanism underlying *miR-277*-mediated rescue of *GMR* > *Aβ42* neurodegenerative phenotype, we used bioinformatics tools like TargetScanFly and BLAST to predict targets of *miR-277*. It is known that miRNAs carry out their function by targeting the 3′UTR of their target mRNAs and thereby regulate gene expression. TargetScanFly predicts the targets of fly miRNA by matching the seed sequence (consensus sequence) of 3’untranslated region of the miRNA and their target mRNA [77] (http://www.targetscan.org/fly_72/). TargetScanFly predicted the proapoptotic factor- *head involution defective (hid)* as one of the mRNA targets with 0.24 P_CT_ (Probability of conserved targeting) score and eight complementary consensus nucleotides (Supplementary Figure 3). Other software tools like DIANA and PicTar also predicted *hid* as one of the targets of *miR-277* with the scores of 0.998 and 18, respectively. Additionally, BLAST tool predicted three different unique consensus sequences that were complementary between *miR-277* and *hid*. Hence, we employed a genetic approach in order to validate whether *hid* and/or other proapoptotic factors is/are the target(s) of *miR-277*.

**Fig. 7:**
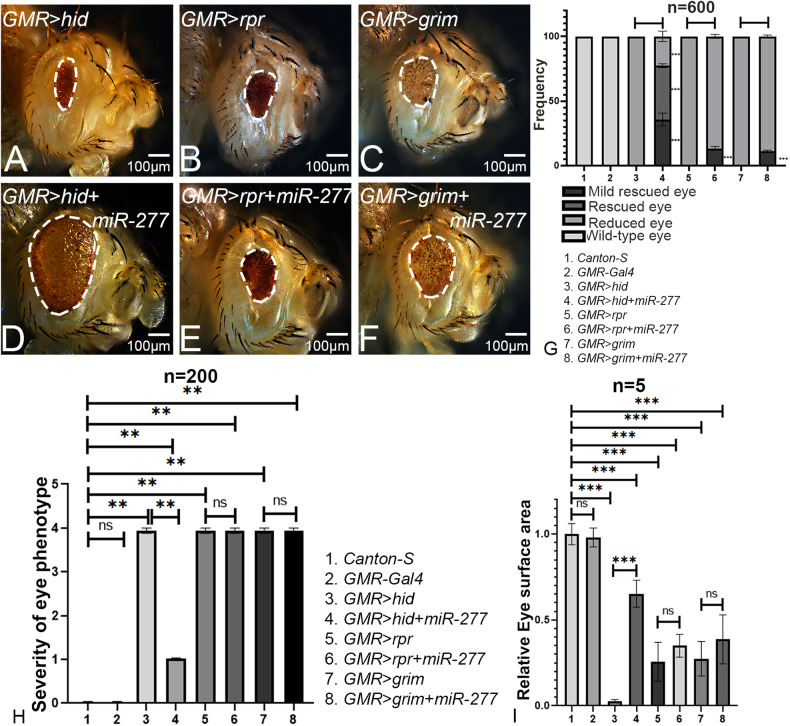
Gain-of-function of *miR-277* rescues the *hid-*mediated cell death. **A** *GMR*>*hid,* **B** *GMR*>*rpr,* and **C** *GMR*>*grim* show reduced eye phenotype due to caspase-mediated cell death. **D** *GMR>hid+ miR-277* results in significant rescue as compared to *GMR>hid* flies. **E** *GMR>rpr+ miR-277* and **F** *GMR>grim+ miR-277* do not show a significant change in eye phenotype as compared to their respective controls **B** *GMR>rpr* and **C** *GMR>grim*. **G** Bar graph shows frequency as average between three repetitions. Two hundred flies were counted per repetition (200 × 3 = 600) to calculate the frequency for each genotype (1. *Canton-S*, 2. *GMR-Gal4*, 3. *GMR>hid*, 4. *GMR>hid+ miR-277*, 5. *GMR>rpr* 6. *GMR>rpr+ miR-277*, 7. *GMR>grim*, 8. *GMR>grim+ miR-277*). Statistical analysis was performed using the student’s *t* test for independent samples. **H** Quantitative analyses of severity score of neurodegenerative phenotypes(s) in eye. Flies from each genotype were randomly selected for scoring according to criteria described in the methods section. Comparisons were made using non-parametric: Mann–Whitney *t* test. **I** Quantitative analyses of area of the eye. The surface area of the eye (within white dotted line) was calculated using Image J. Statistical analysis was performed using the student’s t-test for independent samples. The surface area of *GMR>hid+ miR-277* is significantly rescued (*n* = 5; *p* = 0.0000000073) as compared to *GMR>hid*. Error bars show standard error of mean (mean ± SEM). The orientation of all imaginal discs is identical with posterior to the left and dorsal up. Scale bar = 100 μm.

Gain-of-function of *hid*, *rpr,* and *grim* in the developing eye by using *GMR-Gal4* driver results in a strong phenotype of the highly reduced eye (Fig. 7A–C, G–I). We investigated if gain-of-function of *miR-277* can rescue the reduced eye phenotype of *GMR*>*hid*, *GMR*>*rpr,* and *GMR*>*grim*. Surprisingly, gain-of-function of *miR-277* in the *GMR>hid+miR-277* background exhibit significant phenotypic rescue with frequency (n = 600, 246/600 = 41%) respectively (Fig. 7D, G, H, I). However, gain-of-function of *miR-277* in *GMR>rpr+miR-277* (Fig. 7E, G, H, I) and *GMR>grim+miR-277* (Fig. 7F–I) did not show any rescue. This result ruled out the possibility that the other proapoptotic factors: *rpr* and *grim* being the targets of *miR-277*. Hence, gain-of-function of *miR-277* in the *GMR>hid* flies significantly suppressed the eye degenerative phenotype (Fig. 7H) and increased the surface area of the eye (Fig. 7I), further validating our finding that *hid* is the target of *miR-277*.

Additionally, we also tested ROS by DHE staining in *GMR*>*hid* and observed high levels of ROS puncta (Supplementary Figure 4A). Interestingly, number of ROS puncta significantly reduced when *miR-277* was misexpressed (*GMR*>*hid+miR-277*; Supplementary Figure 4B) as compared to the *GMR*>*hid* alone (Supplementary Figure 4A). The loss-of-function of *miR-277* in *GMR*>*hid* background either by using *miR-277* mutant (*GMR>hid +miR-277* mutant; Supplementary Figure 4C) or Df(3 R)*miR-277*KO (*GMR>hid* + Df(3 R)*miR-277*KO; Supplementary Figure 4D) show significantly increased number of ROS puncta. This suggests that *miR-277* downregulate ROS production in *GMR*>*hid* background.

### *drice* and *Dark* are not the targets of *miR-277*

**Fig. 8:**
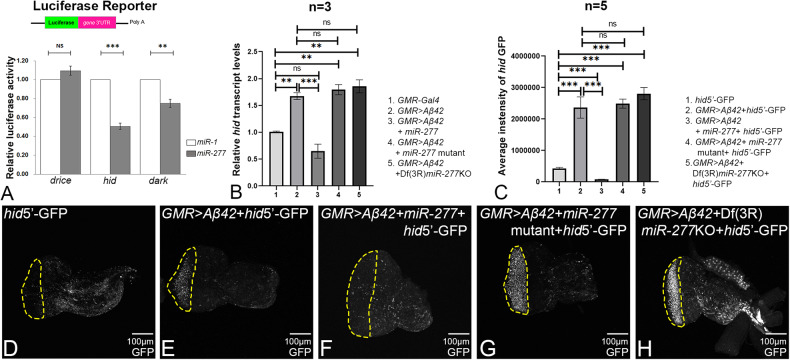
*hid* is the target of *miR-277*. **A** In vitro dual-luciferase reporter assays of miR-mRNA interactions in S2 cell. The mean ± SEM of the relative luciferase expression ratio (firefly luciferase/Renilla luciferase, Luc/R-luc) was calculated for four biological replicates and compared with the control miRNA, miR-1. Statistical analysis was performed using student’s *t* test for independent samples. The results of dual-luciferase reporter assays of target 3′UTR of *drice*, *hid*, and *Dark* have showed that *miR-277* can efficiently target *hid* and *Dark* except for *drice*. **B** Relative expression of *hid* at the transcriptional level using quantitative PCR (qPCR) in genotypes (1. *GMR-Gal4* 2. *GMR* > *Aβ42*, 3. *GMR* > *Aβ42+ miR-277*, 4. *GMR* > *Aβ42+ miR-277* mutant, 5. *GMR* > *Aβ42* + Df(3 R)*miR-277*KO). Triplicate was used for the calculation. Statistical analysis was performed using student’s *t* test for independent samples. *hid transcript* levels were significantly downregulated in *GMR* > *Aβ42+ miR-277* (*n* = 3; *p* = 0.001) whereas slightly increased in *GMR* > *Aβ42+ miR-277* mutant (*n* = 3; *p* = 0.14) and *GMR* > *Aβ42* + Df(3 R)*miR-277*KO) (*n* = 3; *p* = 0.1) as compared to *GMR* > *Aβ42*. **C** Bar graph represents average intensity of *hid* GFP levels within yellow dotted line, region of interest, of five eye imaginal discs per genotype (*n* = 5) (1. *hid5’-*GFP, 2. *GMR* > *Aβ42+ hid5’-*GFP, 3. *GMR* > *Aβ42+ miR-277+ hid5’-*GFP, 4. *GMR* > *Aβ42+ miR-277* mutant*+ hid5’-*GFP, 5. *GMR* > *Aβ42* + Df(3 R) *miR-277*KO*+ hid5’-*GFP). Statistical analysis was performed using student’s *t* test for independent samples. *hid* GFP reporter is significantly downregulated in *GMR* > *Aβ42+ miR-277*+ *hid*5’-GFP (*n* = 5; *p* = 0.002) whereas slightly upregulated in *GMR* > *Aβ42+ miR-277* mutant+ *hid*5’-GFP (*n* = 5; *p* = 0.75) and *GMR* > *Aβ42* + Df(3 R)*miR-277*KO+ *hid*5’-GFP (*n* = 5; *p* = 0.29) as compared to *GMR* > *Aβ42*+ *hid*5’-GFP. Error bars show standard error of mean (mean ± SEM), and symbols above the error bar signify as ***p value < 0.001, **p value < 0.01, *p value < 0.05, and not significant (ns), *p* value > 0.05 respectively. **E** *GMR* > *Aβ42*+ *hid*5’-GFP results in elevated levels of *hid*-GFP levels as compared to the wild-type (**D**) *hid*5’-GFP. **F** *GMR* > *Aβ42+ miR-277*+ *hid*5’-GFP results in the significant downregulation of *hid*-GFP levels, whereas **G** *GMR* > *Aβ42+ miR-277* mutant+ *hid*5’-GFP (**H**) *GMR* > *Aβ42* + Df(3 R)*miR-277*KO+ *hid*5’-GFP result in the upregulation of *hid*-GFP levels. The orientation of all imaginal discs is identical with posterior to the left and dorsal up. Scale bar = 100 μm.

We also explored if other caspases like *drice* or *Dark* get regulated by *miR-277*. We examined the relative luciferase activity of *drice*, *Dark,* and *hid* by luciferase assay in S2 cells. The positive miRNA control used was *miR-1,* which was tagged to different pTub-ffLuc-3′UTR constructs. The value of relative luciferase activities of different pTub-ffLuc-3′UTR constructs with *miR-277* was calculated from the normalization of the luciferase activities of control: *miR-1*. If any of the caspases are the target of *miR-277*, it will bind to the 3′UTR of its target mRNA, degrades the target mRNA, and hence will exhibit less luciferase activity. The relative luciferase activity of *hid* with *miR-277* was significantly reduced by 50% as compared to the positive control (Fig. 8A). The relative luciferase activity of *dark* with *miR-277* was significantly reduced by 25% as compared to the positive control (Fig. 8A). Whereas the relative luciferase activity of *drice* with *miR-277* was slightly increased as compared to the positive control (Fig. 8A). Hence, luciferase activity suggested that *hid* is the target of *miR-277* and not the other caspases.

### *hid* transcript levels are downregulated by *miR-277*

We investigated *hid* transcript levels by qPCR approach. The *hid* transcript levels were significantly increased by ~1.75-fold in *GMR* > *Aβ42* eye imaginal discs as compared to the control *GMR-Gal4* eye imaginal discs (Fig. 8B). *hid* mRNA levels were significantly downregulated by ~2.3-fold in *GMR* > *Aβ42+ miR-277* eye imaginal discs as compared to the *GMR* > *Aβ42* alone (Fig. 8B). However, loss-of-function of *miR-277* in *GMR* > *Aβ42* background by *miR-277* mutant (*GMR* > *Aβ42 +miR-277* mutant) and Df(3 R)*miR-277*KO (*GMR* > *Aβ42* + Df(3 R)*miR-277*KO) resulted in ~1.8 and ~1.9-fold increase in *hid* mRNA levels respectively as compared to the control *GMR-Gal4* (Fig. 8B). Hence, our results strongly suggest that *miR-277* ameliorates Aβ42-mediated neurodegeneration by post-transcriptionally regulating *hid* expression in *GMR* > *Aβ42* flies.

**Fig. 9:**
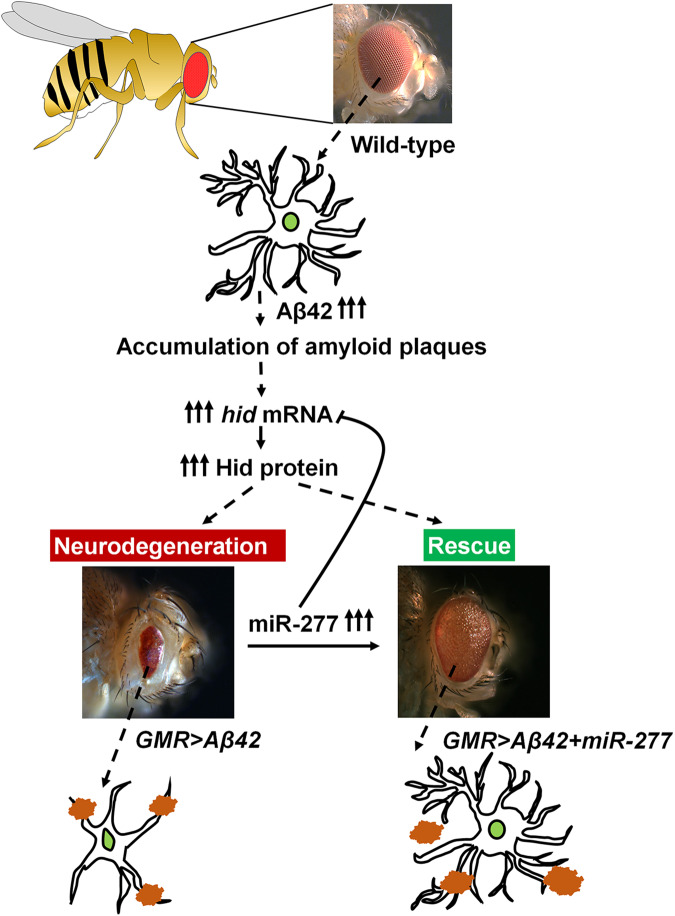
*miR-277* ameliorates the neurodegenerative phenotype of GMR > Aβ42 fly eye. Misexpression of Aβ42 (*GMR* > *Aβ42* flies) results in the accumulation of amyloid plaques and causes aberrant activation of caspase Hid resulting in neurodegenerative adult eye phenotype. Misexpression of *miR-277* in the background of *GMR* > *Aβ42* (*GMR* > *Aβ42+ miR-277*), targets 3′UTR of *hid* mRNA, silences its expression, and hence rescues the reduced eye phenotype observed in *GMR* > *Aβ42* flies.

To validate if *hid* is the target of *miR-277*, we employed *hid*5’F-WT-GFP reporter, which was generated to characterize the role of E2F binding site (present in 5′ ends of *hid*) in *hid* transcription [43]. A 2.2Kb fragment containing the *hid* 5′ E2F binding site was cloned into the pH-stinger GFP vector to generate *hid*5’F-WT GFP transgenic line [43, 78]. The *hid* GFP intensity within the yellow dotted line was calculated from five imaginal discs of each genotype and was used for statistical analysis (Fig. 8C–H). Minimal levels of GFP reporter expression were observed in wild-type *hid*5’F-WT eye-antennal imaginal discs (Fig. 8C, D) [43]. In comparison to the *hid*5’F-WT GFP control (Fig. 8C, D), ~2.5-fold high levels of *hid*-GFP were observed in *GMR* > *Aβ42* eye discs (Fig. 8C, E). Gain-of-function of *miR-277* in the *GMR* > *Aβ42* (*GMR* > *Aβ42+miR-277*) background (Fig. 8C, F) exhibits significant downregulation of *hid*-GFP intensity as compared to *hid*5’F-WT GFP control and *GMR* > *Aβ42* alone (Fig. 8C–E). Loss-of-function of *miR-277* in *GMR* > *Aβ42* flies using *miR-277* mutant (*GMR* > *Aβ42+miR-277* mutant; Fig. 8C, G) and Df(3 R)*miR-277*KO (*GMR* > *Aβ42* + Df(3 R)*miR-277*KO; Fig. 8C, H), showed upregulation of *hid* GFP reporter intensity as compared to the *hid*5’F-WT GFP (Fig. 8C, D). Hence, overexpression of *miR-277* in the background of *GMR* > *Aβ42* (*GMR* > *Aβ42+ miR-277*), targets 3′UTR of *hid* mRNA, silences its expression, and hence rescues the reduced eye phenotype seen in *GMR* > *Aβ42* flies (Fig. 9).

## Discussion

AD, a progressive neurodegenerative disorder, is caused by the accumulation of amyloid plaques and intracellular neurofibrillary tau tangles (NFTs). As per the Amyloid cascade hypothesis, the formation and accumulation of amyloid plaques and NFTs initiate other biochemical changes like oxidative stress, synaptic dysfunction due to aberrant signaling that eventually leads to neuronal cell death [79–81]. However, the mechanism(s) underlying AD-mediated neurodegeneration is not been fully understood [26, 82]. To understand the pathophysiology of AD, many animal models have been generated, including *Drosophila melanogaster*, which has proven very valuable due to the genetic conservation, ease of handling, and the large genetic repository and experimental tools [12, 13, 25]. Human Aβ42 is ectopically misexpressed in differentiating retinal neurons of fly and mimics AD-like neuropathology [17]. *Drosophila* AD models allow us to explore and test the signaling pathways involved in AD by genetic approaches. Numerous signaling pathways like the JNK pathway, Hippo pathway, caspases, GSK pathway etc. are aberrantly activated or dysregulated in AD [17, 83, 84]. Besides, members of these pathways involved in AD also cross-talk with each other making the disease even more complicated.

In eukaryotic systems, microRNAs (miRNAs) are highly conserved and are considered as essential components of gene regulatory networks. miRNAs play crucial roles in the regulation of gene expression and are implicated in various biological processes, including development, metabolism, and disease. miRNAs significantly affect signaling pathways and cause alterations of cellular signaling that can impact human diseases [85, 86]. We identified a highly conserved *miR-277*, as one of the genetic modifiers, which can rescue the Aβ42-mediated neurodegeneration using *Drosophila* eye model. *miR-277* expression is enriched in larval brain, gut, fat body, and adult head, brain, eyes, gut, fat body (https://flybase.org/reports/FBgn0262419.html). The mechanism(s) underlying the effects of *miR-277* targets involved in AD is still unknown.

In AD, synaptic dysfunction occurs due to neuronal cell death and impaired axonal targeting [87, 88]. Using the *Drosophila* eye model, we found that gain-of-function of *miR-277* restores the axonal targeting defects observed in AD [16, 17]. Additionally, *miR-277*-mediated rescue is not restricted to morphological rescue only but it also promotes functional rescue as evidenced from behavioral assays like the eclosion rate and rescue of climbing defects. The *Drosophila* AD model shows robust cell death that is rescued by the gain-of-function of *miR-277*. Moreover, AD is a progressive neurodegenerative disease where the neurodegenerative phenotype progressively worsens with time, such as in pupal retina, photoreceptors are fused due to excessive cell death. However overexpression of *miR-277* restores the hexagonal structure of ommatidia and a number of pigment cells by inhibiting the apoptosis present in *GMR* > *Aβ42* pupal retina. Recently, the anti-apoptotic function of *miR-277* has been reported where gain-of-function of *miR-277* rescues the tumor suppressor gene, lethal giant larvae (*lgl)*, mutant clones in third instar larvae, which would otherwise be eliminated due to cell death [89], and *miR-277* is also expressed in hub cells and may contribute to their protection [90, 91].

Furthermore, to discern the molecular genetic mechanism(s) for *miR-277* neuroprotective function, we screened for its target mRNA and identified *hid*, a proapoptotic gene, as one of its targets. Earlier, we have shown that aberrant activation of evolutionarily conserved JNK signaling pathway induces *hid* expression, which triggers cell death response seen in Aβ42-mediated neurodegeneration [17]. In *GMR* > *Aβ42* background there is a significant decrease in *hid* transcript levels, as evident from *hid*-GFP reporter studies and qPCR assays. However, gain-of-function of *miR-277* in *GMR* > *Aβ42* significantly reduces the *hid* transcript levels. Based on our results from *hid* transcript level studies as well as luciferase reporter assay, we propose a model where *miR-277* binds to 3′UTR of *hid* and degrades the expression of *hid* transcript (Fig. 9). Hence, we show here for the first time that *miR-277* achieves its neuroprotective function by downregulating *hid* transcript levels thereby ameliorating Aβ42-mediated neurodegeneration. *miR-277* has also been implicated in neurodegenerative disease like Fragile X-associated tremor/ataxia syndrome (FXTAS) [92]. However, its role in other neurodegenerative disorders like ALS, Parkinson’s, Huntington, etc is not yet determined. Further studies on the regulatory network and downstream targets of *miR-277* will deepen our understanding of its biological significance and potential implications in human health and other neurodegenerative diseases. Additionally, microRNAs (miRNAs), which serve as invaluable tool for manipulating target gene expression [93, 94], have gained attention as potential biomarkers in various diseases, including neurodegenerative disorders like AD [95]. MiRNAs, carried by exosomes and microparticles in the blood and exhibiting greater stability than mRNAs, are valuable diagnostic biomarkers to enhance the accuracy of AD diagnoses [35, 96]. Insights into the roles of miRNAs in AD, particularly through affecting the signaling pathways, can make miRNAs attractive tools for novel therapeutic approaches.

Mutations affecting three proapoptotic genes: *hid, rpr* and *grim,* nearly completely eliminate the process of apoptosis during development [97]. However, research suggests that the overexpression of *hid* leads to cell death in various tissues of transgenic animals, as well as in cultured insect and mammalian cells [98]. Previous study demonstrates that role of *hid* as an inducer of cell death in *Drosophila* is conserved in mammalian cells, suggesting the possible existence of a mammalian homolog of this crucial apoptosis regulator [97]. When co-transfected with certain inhibitors, apoptosis is significantly reduced, indicating that Bcl2-type anti-apoptotic genes can inhibit Hid-induced apoptosis in mammalian cells. The inhibition of Hid’s proapoptotic activity by DIAP1 in mammalian cells mirrors a similar regulatory process as in insect cells, and the involvement of a mammalian IAP, XIAP, further indicates the evolutionary conservation of IAP-mediated inhibition of Hid activity [97]. *Drosophila* RHG proteins are functional counterparts to the mammalian Smac/DIABLO proteins, and they work by binding to and reducing the levels of IAP [99, 100]. Hence, the presence of an apoptosis pathway activated by *hid* in mammalian cells, regulated by conserved molecular components, strongly suggests the existence of a vertebrate *hid* counterpart.

Based on the conserved seed sequence, the human ortholog of *miR-277* is hsa-miR-3660 (https://www.mirbase.org/). hsa-miR-3660 is expressed in the nervous system (brain and cortex) and reproductive system (testis and uterus) as shown in RNA seq data (https://www.genecards.org/cgi-bin/carddisp.pl?gene=MIR3660). It has been shown by GWAS studies that hsa-miR-3660 is implicated in human diseases such as attention deficit hyperactivity disorder [101], rheumatic heart disease [102], and lung cancer [103–105]. Moreover, it has been shown that hsa-miR-3660 is dysregulated in pseudoexfoliation glaucoma (PEXG) [106]. However, its role in Alzheimer’s Disease has not been shown to date. A multiple sequence alignment analysis has identified a putative binding site for hsa-miR-3660 within the 3′ untranslated region (3′UTR) of the gene *hid*. It is possible that hsa-miR-3660 could bind to the mammalian component of *hid* and degrade it. One of the potential targets predicted of hsa-miR-3660 is associated with the CARD domain (https://www.targetscan.org/vert_80/). The CARD domain, which stands for Caspase Activation and Recruitment Domain, is a protein module involved in initiating and regulating various signaling pathways, particularly those related to apoptosis (programmed cell death) and inflammation [107, 108]. The CARD domain in caspase 2 and caspase 9, initiator caspases, facilitate the formation of protein complexes involved in the apoptotic signaling cascade [107, 108]. In AD, the dysregulation of caspase 2 and caspase 9 activation may contribute to the loss-of neurons and the progression of the disease [107]. Hence, the identification of specific targets regulated by hsa-miR-3660 holds significant promise for advancing therapeutic strategies for AD. Further exploration of hsa-miR-3660 in AD mammalian model systems and AD patient’s brain samples and biofluids can help shed light on the etiology of AD or potential role of hsa-miR-3660 (an ortholog of fly *miR-277*) as a biomarker and as a druggable target of AD. In the future, hsa-miR-3660 inhibitor, can be employed to specifically bind to and inhibit the activity of endogenous hsa-miR-3660.

### Supplementary information

Supplementary Data

Checklist

## Data Availability

All data generated or analyzed during this study are included in this published article [and its supplementary information files].
