# Supplementary material for: miR-277 targets the proapoptotic gene-hid to ameliorate Aβ42-mediated neurodegeneration in Alzheimer’s model
Source: Cell Death Dis. 2024 Jan 18;15(1):71. doi: 10.1038/s41419-023-06361-3 (PMC10796706; doi:10.1038/s41419-023-06361-3)
Supplement: Supplementary file 1 — Supplementary Data [file 41419_2023_6361_MOESM1_ESM.docx]

**Supplementary Figures**


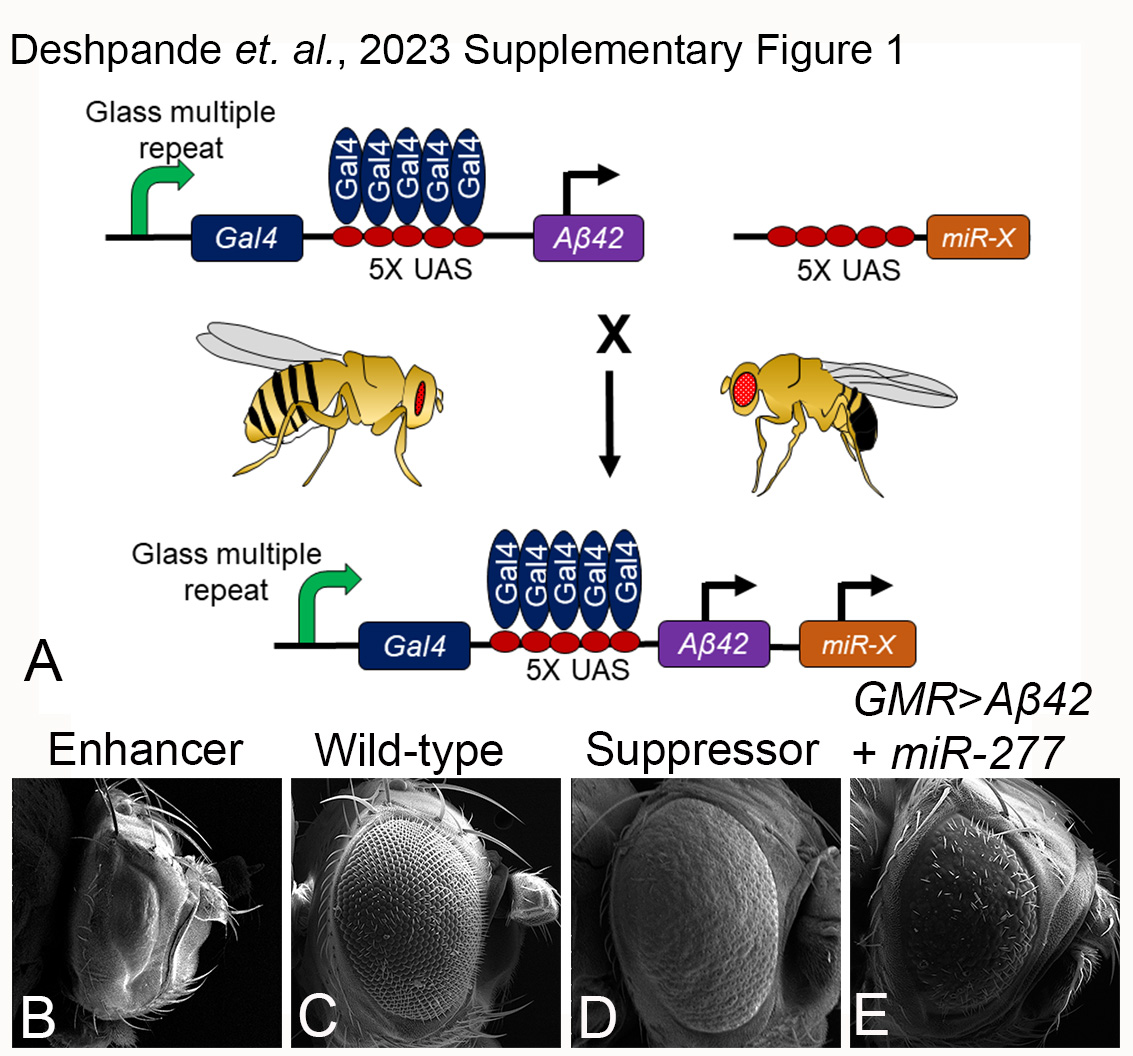


**Supplementary Figure 1: Strategy for forward genetic screening to identify candidate miRNAs as genetic modifiers of Aβ42- mediated neurodegeneration.** Flies expressing human Aβ42 in the developing eye under the control of the GMR-Gal4 driver exhibit small, rough eye phenotype. They were crossed individually to flies with different candidate miRNAs expressed under UAS control (normal eyes). Eye phenotypes were then observed in the progeny and screened for either an enhancer or suppressor of the Aβ42 eye phenotype. In forward genetic screening we identified *miR-277* as the suppressors of Aβ42 eye phenotype (rescue eye phenotype).


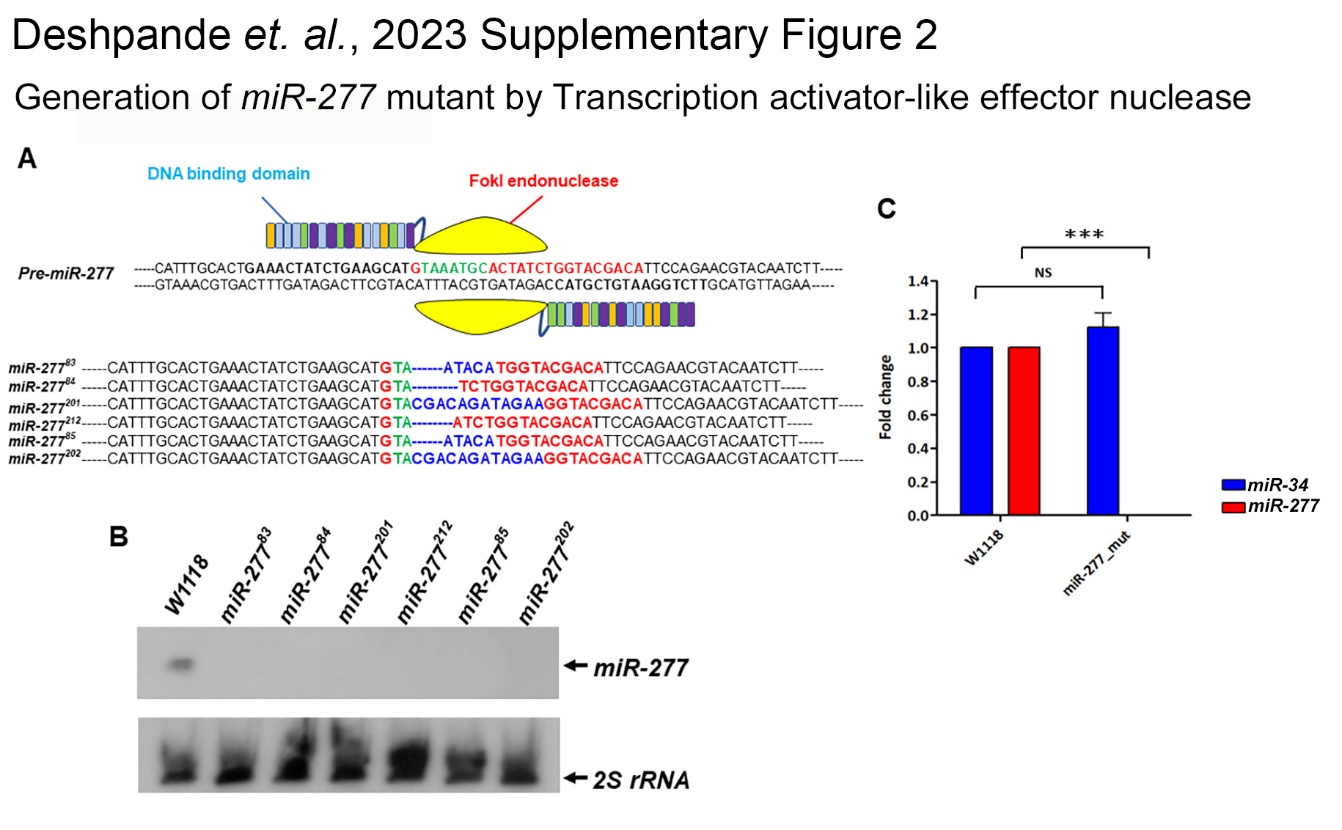
 **Supplementary Figure 2: Generation of *miR-277* mutant line by TALEN.** (A) Schematic of *miR-277*_TALEN pair binding to pre-*miR-277* DNA. The bold was indicated *miR-277*_TALEN binding site (Black), mature *miR-277* (Red) and seed region (green) (top). The small indels of *miR-277* mutant lines were showed in the blue bold (bottom). (B)  The expression of mature *miR-277* RNA was validated in the wild type W1118 and six *miR-277* mutant lines by northern blotting. (C) Quantitative polymerase chain reaction analysis of *miR-277* and *miR-34* expression in wild type W1118 and *miR-277* mutant line. Data are presented as the mean ± standard deviation, n=3. *** p-value <0.001 vs. the W1118. U6 was used for internal control.


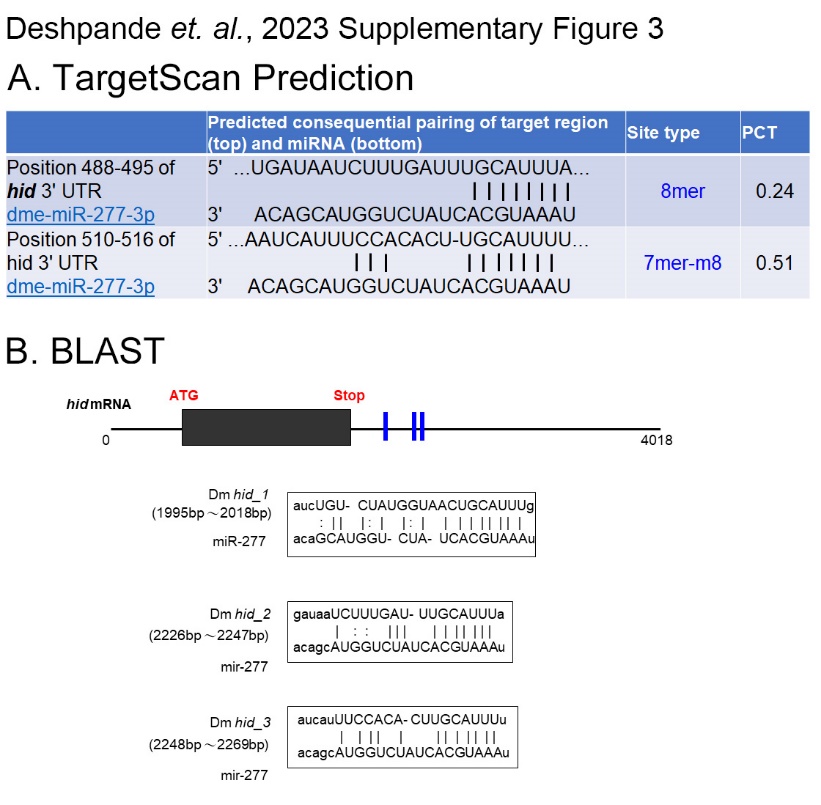
 **Supplementary Figure 3**: ***miR-277* regulates *hid* 3’UTR.** (A) TargetScanFly (http://www.targetscan.org/fly_72/) predicted *hid* as one of the targets of *miR-277* based on the seed sequence. (B) Schematic representation of the mRNA sequence of *hid* from *Drosophila melanogaster*. According to the web BLAST of NCBI (https://blast.ncbi.nlm.nih.gov/Blast.cgi) analysis between the 3’UTR of *hid* mRNA and *miR-277*, three candidate target sites can be recognized by the seed region of *miR-277*. Alignment of *miR-277* miRNA with the predicted target sites from the *hid* 3’UTR are shown below.


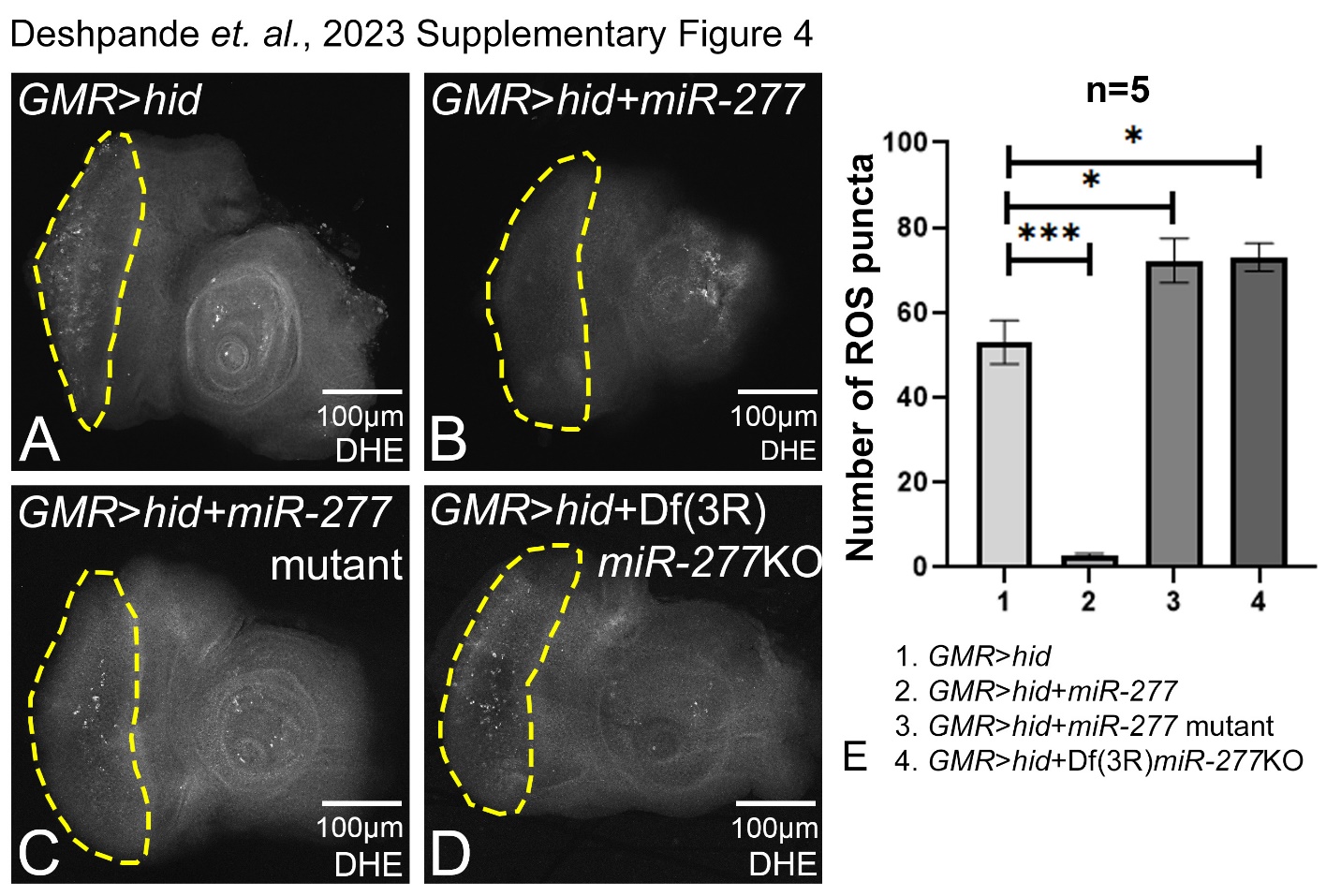
 **Supplementary Figure 4: Modulation of *miR-277* in *GMR>hid* downregulates ROS production.** Dihydroethidium (DHE) is employed to detect ROS produced in cells. (A) *GMR>Aβ42* shows elevated levels of ROS puncta (B) Gain-of-function of *miR-277* in the background of *GMR*>*hid* (*GMR>hid+ miR-277*) results in significant reduction in the ROS production as compared to *GMR*>*hid*. Loss-of-function of *miR-277* in *GMR*>*hid* background using (C) *miR-277* mutant (*GMR>hid+ miR-277* mutant), and (D) Df(3R)*miR-277*KO (*GMR>hid+* Df(3R)*miR-277*KO) results in the increased ROS production. (A-E) ROS puncta were counted within yellow dotted line, the region of interest, for the statistical analysis. We quantified ROS puncta in photoreceptor cells of five eye imaginal discs per genotype (n=5) (1. *GMR>hid*, 2. *GMR>hid+ miR-277*, 3. *GMR>hid+ miR-277* mutant, 4. *GMR>hid +* Df(3R)*miR-277*KO). Statistical analysis was performed using student’s t-test for independent samples. *GMR>hid+miR-277* exhibits significant reduction in ROS puncta as compared to *GMR>hid* (n=5; p=0.00052) whereas *GMR> hid + miR-277* mutant (n=5; p=0.03) and *GMR>hid+* Df(3R)*miR-277*KO (n=5; p=0.02) show significant increase in ROS puncta as compared to *GMR>hid*. Error bars show standard error of mean (mean ± SEM), and symbols above the error bar signify as *** p-value <0.001, ** p-value <0.01, * p-value <0.05, and not significant (ns), p-value >0.05 respectively. The orientation of all imaginal discs is identical with posterior to the left and dorsal up. Scale bar= 100 μm


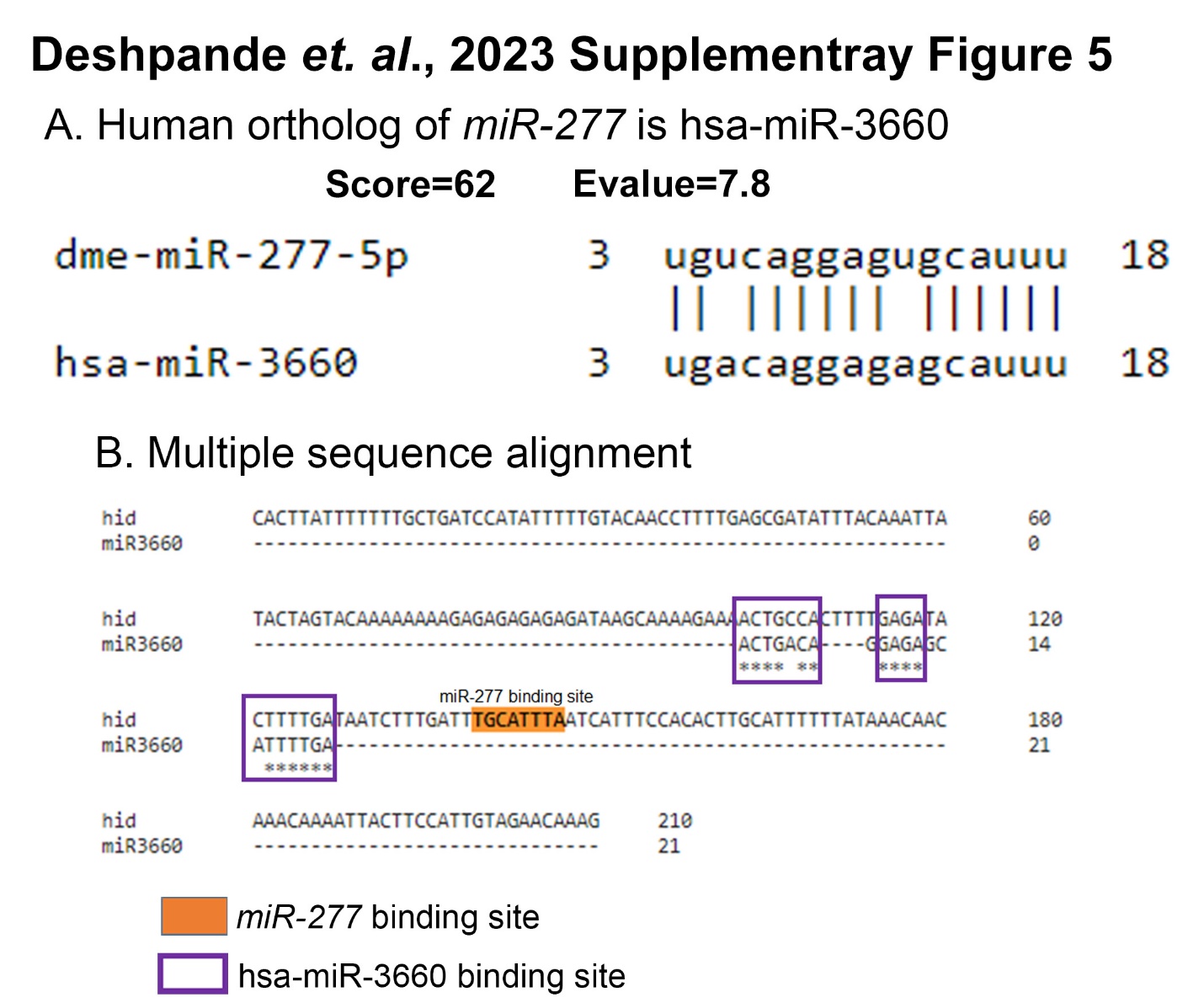


**Supplementary Figure 5: A. Human ortholog of *miR-277* is hsa-miR-3660:** miRDB (<https://mirdb.org/>) predicts the orthologs of miRNAs. Dme-*miR-277*-5p and *hsa-miR-3660* shows conserved nucleotide sequence with score=62. **B. Multiple sequence alignment:** Clustal omega predicts the hsa-miR-3660 binding site at 3’UTR of *hid.*
